# Comparison of RNA- and DNA-based 16S amplicon sequencing to find the optimal approach for the analysis of the uterine microbiome

**DOI:** 10.1038/s41598-025-00969-5

**Published:** 2025-05-16

**Authors:** Antonia I. Dyroff, Álvaro López-Valiñas, Humberto B. Magalhaes, Giorgia Podico, Igor F. Canisso, Carmen Almiñana, Stefan Bauersachs

**Affiliations:** 1 Institute of Veterinary Anatomy, Vetsuisse Faculty Zurich, University of Zurich, Lindau (ZH), Switzerland; 2 College of Veterinary Medicine, University of Illinois Urbana-Champaign, Urbana, IL USA; 3 Department of Reproductive Endocrinology, University Hospital Zurich, Zurich, Switzerland

**Keywords:** *Equus caballus*, Reproductive pathology, Endometrial health, Bacterial viability, Microbial community analysis, Microbiome, Metagenomics, Urogenital reproductive disorders, Infertility, Sequencing, DNA sequencing, Next-generation sequencing, Reproductive biology

## Abstract

Studies in humans and large animals indicate a relationship between the uterine microbiome composition and endometrial receptivity. Despite many studies have been performed, the analysis of the uterine microbiome remains challenging due to the very low microbial biomass. Studies in other biological systems showed that RNA-based microbiome analysis complements DNA-based results and provides information about active bacteria in a sample. Thus, the aim of this study was to establish a highly sensitive and specific 16S rRNA gene V3-V4 amplicon PCR from equine uterine cytobrush samples and to compare DNA- and RNA-based 16S rRNA microbiome analysis. An optimized 16S rRNA gene V3-V4 amplicon PCR protocol from equine uterine cytobrush samples was developed, which was able to detect less than 38 bacterial genome copies using a bacterial DNA community standard. For the RNA-based amplicon generation protocol starting from cDNA, at least a 10-fold higher sensitivity was estimated compared to DNA-based approach. The comparison of using RNA and DNA isolated from the same uterine cytobrush samples as input for 16S V3-V4 amplicon sequencing revealed a much higher number of amplicon sequence variants as well as taxonomic units for the RNA-based approach. This resulted in significant differences in alpha (Simpson, Chao1) and beta diversity between RNA- and DNA-based analysis. Differential abundance analysis revealed significant differences between DNA and RNA samples at all taxonomic levels. Despite these differences, the overall microbiome composition was similar between the paired DNA and RNA samples. Many differences were probably found due to the higher sensitivity of the RNA-based approach. Furthermore, the DNA-based analysis is biased by the rRNA gene copy numbers (1–21), and the RNA-based analysis by the number of ribosomes per cell, which was reflected in the differences in the microbiome composition between the approaches. In addition, the results suggested that the DNA-based analysis is detecting cell-free bacterial DNA and/or DNA of dead bacteria that could be present in the samples. Altogether, the obtained results indicate advantages of a combined DNA- and RNA-based microbiome analysis, offering complementary and valuable information in the context of fertility-related studies of the uterine microbiome.

**Supplementary Information:**

The online version contains supplementary material available at 10.1038/s41598-025-00969-5.

## Introduction

The advent of next-generation sequencing technologies finally enabled a global assessment of the uterine microbiome and solved finally the discussion about the historical dogma of the sterile uterus^1–5^. A correlation of the female reproductive microbiome composition with reproductive success or failure at different stages of establishment and maintenance of pregnancy has been shown or suggested in humans and other mammalian species^6–10^. For the uterus, microbial communities are usually analyzed using 16S rRNA gene amplicon sequencing, because of the high background of host DNA and very low microbial DNA amounts^11^. Many studies analyzing the very low biomass uterine bacterial microbiome pointed at the challenges and potential contaminations from various sources during sampling and amplicon generation^4,6,12,13^.

The 16S rRNA gene amplicon sequencing using bacterial DNA as a PCR template (DNA-based) brings several limitations to the conclusions on bacterial microbiome composition. First, bacterial DNA in the sample may originate from both live and dead bacteria. Thus, DNA-based 16S analysis does not accurately reflect the active and living microbiota at the time of sampling^14^. Furthermore, rRNA gene copy numbers vary from 1 to 15 (21, https://rrndb.umms.med.umich.edu/) per genome depending on the bacterial phyla^15^. If uncorrected, this leads to underestimation of bacterial phyla with low and overestimation of phyla with high copy numbers^15^. In contrast, RNA-based 16S rRNA amplicon sequencing detects active bacteria^14^, but also has some bias due to differences in 16S rRNA copy numbers, i.e., in the number of ribosomes per cell, particularly between very small and larger bacteria, as well as bacteria with varying growth rates^16,17^. Concerning the analysis of the very low bacterial biomass uterine microbiome, the RNA-based approach is supposed to be more sensitive given the much higher number of ribosomes per bacterial cell (e.g., *E. coli* with ~ 25,000 per cell^18,19^ compared to rRNA gene copy numbers per bacterial genome (1–15)^15^. Thus, RNA-based sequencing could enable the detection of rare taxa in low bacterial biomass samples which are hardly detectable with the DNA-based approach.

Considering the above-mentioned limitations, the aim of the present study was to establish a highly sensitive and specific 16S rRNA gene V3-V4 amplicon sequencing protocol for characterizing the equine uterine microbiome from low bacterial biomass obtained from uterine cytobrush samples. The use of the cytobrush for uterine sampling is a common routine practice in the diagnostics of endometritis in the mare^20^, and it is the preferred method since it is non-invasive and less harmful compared to a biopsy. For this purpose, DNA-based vs. RNA-based 16S rRNA gene V3-4 amplicon sequencing starting from DNA and RNA derived from the same uterine cytobrush sample was compared. We hypothesized that the RNA-based approach offers higher sensitivity, resulting in a higher diversity of bacterial phyla with lower abundance. Furthermore, the comparison aimed at identifying differences between the results of the two approaches and their implications for uterine microbiome analysis in consideration of the microbiome-uterine receptivity relationship.

## Material & methods

### Collection of uterine cytobrush samples

Uterine cytobrush samples were collected from 14 mares owned by the University of Illinois Urbana-Champaign or client-owned mares presented at the Veterinary Hospital. All experimental protocols were approved by the University of Illinois Institutional Animal Care and Use Committee (IACUC approval numbers 21237 and 21238). All methods were carried out in accordance with relevant guidelines and regulations. The study was carried out in compliance with the ARRIVE guidelines (https://arriveguidelines.org/).

The mares were restrained in the stock, the tail was wrapped and secured to the side. The rectum was emptied, and the perineal area was prepped with iodine solution and warm water. Transrectal palpation and ultrasound were performed to determine the stage of the estrous cycle. The ovarian structures and their size and the presence of uterine edema and fluid were recorded. The fluid was measured at the base of one of the two uterine horns using the caliper tools of the ultrasound machine. Once determined in estrus, the cytobrush samples were collected. One double-guarded cytobrush (Minitube, Tiefenbach, Germany) was introduced into the uterus and rolled on the uterine surface for 15–30 s. The cytobrush was then removed and placed in a cryotube containing 350 µl of RLT Plus lysis buffer (Qiagen, Germantown MD, USA) with dithiothreitol (DTT; 20 µl of 2 M DTT per 1 ml RLT Plus buffer); the cytobrush was left in the tube and rolled for 20–30 s and then discarded. The cryotube with the lysis buffer was frozen in liquid nitrogen and stored at − 80 °C until the isolation of RNA and DNA.

### Extraction of DNA and RNA

DNA and RNA were isolated with the AllPrep DNA/RNA/miRNA Universal Kit (Qiagen AG, Hombrechtikon, Switzerland). To each sample (lysate in 350 µl RLT Plus lysis buffer), additional 250 µl of RLT Plus buffer were added before shaking on an IKA^®^ Vibrax VXR basic, Type VX 2 E (IKA Werke GmbH and Co. KG, Staufen im Breisgau, Germany) at 1500 rpm for 10 min at room temperature. The subsequent steps were performed according to the manufacturer’s instructions using the protocol of “Simultaneous Purification of Genomic DNA and Total RNA, including miRNA, from cells”. Elution of RNA and DNA was carried out with 2 × 30 µl of RNase-free water and 2 × 50 µl elution buffer (EB), respectively, with an incubation for 5 min before centrifugation. The concentration and purity of RNA and DNA were measured with a NanoDrop One spectrophotometer (ThermoFisher Scientific, Reinach, Switzerland) and the QuantiFluor^®^ RNA System and QuantiFluor^®^ ONE dsDNA System with a Quantus fluorometer (Promega AG, Dübendorf, Switzerland) according to the manufacturer’s protocols. The quality of the isolated RNA was assessed using the Agilent 2100 Bioanalyzer RNA 6000 Nano assay (Agilent Technologies, Waldbronn, Germany).

### 16S rRNA gene V3-V4 amplicon PCR

All reactions were performed in Eppendorf PCR Tubes 0.2 mL PCR clean, (0030124332, Vaudaux-Eppendorf AG, Basel, Switzerland) in a Mastercycler X50s (6311000010, Eppendorf). Sequences for PCR primers, 12 S rRNA blocking oligonucleotides, and the peptide nucleic acid (PNA) clamp (PNA Bio Inc, Newbury Park (CA), USA) are shown in Table 1. Primers Pro341F and Pro805R sequences were derived from Takahashi S. et al. (2014)^21^. All oligonucleotides were purchased from Integrated DNA Technologies, Inc. (via LubioScience GmbH, Zürich, Switzerland). The positive control used for 16S rRNA gene V3-V4 amplicon PCR contained a mix of bacterial DNA isolated from cultured *Escherichia coli*, *Streptococcus equi*, *Streptococcus equi subsp. zooepidemicus*, *Streptococcus dysgalactiae equisimilis*, *Klebsiella pneumoniae*, *Pseudomonas aeruginosa*, and *Staphylococcus aureus*, prepared and donated by Dr. Joachim Spergser (University of Veterinary Medicine, Vienna). Equal amounts of every DNA sample were added with a final total concentration of the mix of 10 pg/µl. In addition, the positive control contained 20 ng/µl mouse genomic DNA as eukaryotic background genomic DNA (G3091 Mouse Genomic DNA, Promega AG). The ZymoBIOMICS Microbial Community DNA Standard (D6305, Lucerna Chem AG, Luzern, Switzerland) was used for the experiment with the serial dilutions to test the sensitivity of the 16S V3-V4 amplicon PCR. As a negative control, DNA-free water was used (P-020-0003, DNA-free water, PCR-grade, Molzym GmbH & Co. KG, Bremen, Germany or MBD0025-10 × 1.5ML, water, microbial DNA-free, Merck & Cie, Buchs, Switzerland).

**Table 1:** Oligonucleotide sequences.

Oligonucleotide name	Sequence (5’-3’)
Pro341F	NCCTACGGGNBGCASCAG
Pro805R	GACTACNVGGGTATCTAATCC
Pro341F_(1–4)N	CTTTCCCTACACGACGCTCTTCCGATCT(N)_1−4_CCTACGGGNBGCASCAG
Pro805R_(1–4)N	GGAGTTCAGACGTGTGCTCTTCCGATCT(N)_1−4_GACTACNVGGGTATCTAATCC
12 S rRNA PNA PCR clamp	AGTGACTTTAATACCTCTGA
12 S rRNA blocking oligo-amino	GTGGGGTATCTAATCCCAGTTTGGG/3AmMO
12 S rRNA blocking oligo-phosphate	GTGGGGTATCTAATCCCAGTTTGGG/3Phos
TSD501	AATGATACGGCGACCACCGAGATCTACACTATAGCCTACACTCTTTCCCTACACGACGCTCTTCCGATCT
TSD502	AATGATACGGCGACCACCGAGATCTACACATAGAGGCACACTCTTTCCCTACACGACGCTCTTCCGATCT
TSD503	AATGATACGGCGACCACCGAGATCTACACCCTATCCTACACTCTTTCCCTACACGACGCTCTTCCGATCT
TSD504	AATGATACGGCGACCACCGAGATCTACACGGCTCTGAACACTCTTTCCCTACACGACGCTCTTCCGATCT
TSD505	AATGATACGGCGACCACCGAGATCTACACAGGCGAAGACACTCTTTCCCTACACGACGCTCTTCCGATCT
TSD506	AATGATACGGCGACCACCGAGATCTACACTAATCTTAACACTCTTTCCCTACACGACGCTCTTCCGATCT
TSD507	AATGATACGGCGACCACCGAGATCTACACCAGGACGTACACTCTTTCCCTACACGACGCTCTTCCGATCT
TSD508	AATGATACGGCGACCACCGAGATCTACACGTACTGACACACTCTTTCCCTACACGACGCTCTTCCGATCT
Amp_N714	CAAGCAGAAGACGGCATACGAGATGCTCATGAGTGACTGGAGTTCAGACGTGTGCTCTTCCGATC
Amp_N715	CAAGCAGAAGACGGCATACGAGATATCTCAGGGTGACTGGAGTTCAGACGTGTGCTCTTCCGATC
Amp_N716	CAAGCAGAAGACGGCATACGAGATACTCGCTAGTGACTGGAGTTCAGACGTGTGCTCTTCCGATC
Amp_N718	CAAGCAGAAGACGGCATACGAGATGGAGCTACGTGACTGGAGTTCAGACGTGTGCTCTTCCGATC

### Amplicon PCR protocol starting from DNA

In all DNA-based PCRs, 20 ng DNA isolated from cytobrush samples was used in a reaction volume of 12.5 µL. For the experiments to block an unspecific product derived from the equine 12 S mitochondrial RNA gene using the PNA clamp or oligonucleotides blocking the priming site in the 12 S mitochondrial RNA gene, the reaction mixture contained 1x PCR Buffer (–MgCl_2_); 1.5 mM MgCl_2_, DNA‑free; 0.2 mM dNTP mix; 0.2 µM each for primer Pro341F_Illu_1N and Pro805R_Illu_1N; 0.625 U Invitrogen™ Platinum™ Taq DNA Polymerase, DNA-free (16315062, Fisher Scientific AG, Reinach, Switzerland). 12 S rRNA PNA clamp had a final concentration of 0.5 and 1 µM, respectively. The 12 S rRNA blocking oligonucleotides had a final concentration of 0.2 and 0.4 µM, respectively. The thermal cycling conditions for experiments with 12 S rRNA PNA clamp were as follows: initial denaturation at 94 °C for 120 s; 35 cycles of 1st annealing at 68 °C for 10 s, 2nd annealing at 58 °C for 30 s, and extension at 72 °C for 45 s; final extension at 72 °C for 300 s. The PCR reaction conditions for experiments with 12 S rRNA blocking oligonucleotides were identical except for the 1st annealing, which was at 70 °C for 20 s.

The final PCR protocol used to estimate PCR sensitivity and compare RNA- and DNA-based 16S rRNA gene V3-V4 amplicon generation was further optimized. The reaction mixture (12.5 µL) contained 1x PCR Buffer (–MgCl_2_); 2 mM MgCl_2_, DNA‑free; 0.3 mM dNTP mix; 0.2 µM each Pro341F_Illu_(1–4)N and Pro805R_Illu_(1–4)N primer; 0.04 µM each Pro341F and Pro805R primer; 0.6 µM 12S rRNA blocking oligonucleotide with 3’-NH_2_; and 1 U Invitrogen™ Platinum™ Taq DNA Polymerase, DNA-free. The thermal cycling conditions were as described above (12 S rRNA blocking oligonucleotide, 1st annealing at 70 °C for 20 s).

### Amplicon PCR protocol starting from RNA

Synthesis of first-strand cDNA was performed starting from 250 ng total RNA with Maxima H Minus Reverse Transcriptase (EP0752, ThermoFisher Scientific) in 0.5 ml PCR clean Eppendorf^®^ tube. The reaction mixture (20 µL) contained 1X RT Buffer, 0.5 mM dNTP mix; 1 µM Pro805R primer, and 2 µM 12S rRNA blocking oligonucleotide with 3’-NH_2_. After gentle mixing and brief centrifugation, the reactions were incubated at 70 °C for 5 min in a Mastercycler Nexus X2 (Eppendorf). After cooling to 57 °C, 0.5 µl Maxima H Minus Reverse Transcriptase (200 U/µl) was added to each reaction. The vials were immediately put back on the thermal cycler for 1 min, then briefly mixed and centrifuged, and transferred back to the thermal cycler for 30 min at 57 °C. The reaction was terminated by incubation at 85 °C for 5 min. Of the reverse transcription reaction, 1 µl was directly used as a template for 16S rRNA gene V3-V4 amplicon PCR. The PCR reactions were the same as for DNA samples but scaled up to 25 µl reaction volume.

### Agarose gel electrophoresis for analysis and isolation of amplicon PCR products

1% agarose gels were prepared using standard agarose Roti^®^Garose (3810.2, Roth AG, Karlsruhe, Germany) and 50x TAE buffer (2.0 M Tris, 1.0 M acetic acid, 0.05 M EDTA) to separate the amplicon PCR products. GelRed^®^ Nucleic Acid Stain (Biotium, San Francisco, U.S.) was used to stain the DNA, and GeneRuler 100 bp Plus DNA Ladder (SM0323, ThermoFisher Scientific^®^) (50 and 100 ng) as a nucleic acid molecular weight marker. Imaging and quantification were performed with the ChemiDoc MP system (302 nm UV transilluminator) and ImageLab software v.6.1. (Bio-Rad Laboratories). The 16S rRNA gene V3-V4 amplicon products were excised from the gel using x-tracta gel extraction tools (Z722390-100EA, Merck & Cie). The PCR products were purified using the QIAEX II^®^ Gel Extraction Kit (20021, Qiagen AG) according to the manufacturer’s instructions. Isolated PCR products (expected and unspecific products) were sent for identification for Sanger sequencing (https://www.microsynth.com).

### Amplicon sequencing and data analysis

Samples from 14 mares were used for the generation of 16S rRNA gene V3-V4 amplicons. From the same cytobrush sample, RNA and DNA was isolated, obtaining a total of 28 samples, 14 RNA and 14 DNA samples. After isolation of the 16S rRNA V3-V4 product from agarose gels, the concentration was measured with the Quantus^®^ fluorometer. Amplicon barcoding was performed at the Functional Genomics Center Zurich (https://fgcz.ch). A combination of 8 forward (TSD501-508) and 4 reverse indexing primers (Amp_N714, 715, 716, 718, Table 1) was used for the 30 samples (28 cytobrush samples and 2 controls). After barcoding, PCR products were purified using 0.8x magnetic beads (Sera-Mag Select Beads, Cytiva, Marlborough, USA). The quality and quantity of the resulting libraries were assessed using the 4200 TapeStation system (Agilent Technologies). Libraries were normalized to a concentration of 10 nM in Tris-HCl (10 mM, pH 8.5, 0.1% Tween 20) and pooled in equimolar amounts before 2 × 300 bp paired-end sequencing on an Illumina NextSeq2000 instrument according to standard protocols.

### Amplicon sequencing data analysis

The obtained FASTQ files were processed and analyzed with QIIME 2 (version 2024.5) to characterize the microbial community composition^22^. The quality of the raw data was evaluated with the q2-demux plugin. The 5’-ends of the R1 and R2 reads were trimmed including the V3-V4 amplicon PCR primers with q2-cutadapt^23^. Quality filtering, chimeras removed, and sequences truncation at 276 bp for forward and 214 pb for reverse reads were performed using DADA2 (via q2-dada2)^24^, after which amplicon sequence variants (ASVs) were inferred. Furthermore, ASVs resulting from possible PCR contaminants were removed using the VSEARCH method, from the *quality-control exclude-seqs plugin*, against SILVA 132 at 97% similarity, with an 80% identity threshold^25^. Taxonomy was assigned to ASVs with the *qiime feature-classifier ‘classify-sklearn’ plugin*, using the Greengenes 2 database trained for V3-V4 data (clustered at 99% of identity)^26^. Neglectable ASVs with fewer than 5 reads (corresponding to 5–10 counts per million, cpm) in at least 5 samples were removed. However, ASVs with at least 20 reads (20–40 cpm) in 1 sample were recovered, using the qiime feature-table filter-features plugin. The abundance tables at different taxonomic levels were obtained using qiime taxa collapse.

For microbiome diversity analysis, the samples were rarefied to the sequencing depth of 1,118,227, corresponding to the sample with the lowest depth. For the phylogenetic tree representation, ASVs were aligned using mafft^27^, with hypervariable positions masked using qiime alignment mask. Subsequently, trees were constructed with FastTree^28^, and the rooted trees were generated using iTOL^29^. Alpha diversity metrics were calculated by applying the Shannon, Simpson, and Chao1 indices with *qiime diversity* plugins. Significant differences were assessed using a paired t-test for the Shannon index, and the non-parametric Wilcoxon test for paired data for the Simpson and Chao1 indices. Beta diversity metrices were used to evaluate differences in microbial community composition between samples using Jaccard and Bray-Curtis dissimilarity indices. Principal coordinate analysis (PCoA) was performed with *qiime core-metrics* and a PERMANOVA pairwise test was conducted to assess Beta diversity differences between groups using *qiime diversity beta-group-significance*. Differential abundance analysis was performed with Analysis of Composition of Microbiomes (ANCOM)^30,31^ using ALDEx analysis^32^. All graphs and statistical analysis in this study were performed using RStudio (2024.4.1.748)^33^, with the ‘ggplot2’, ‘dplyr’, and ‘plotly’ packages.

## Results

### Establishment of 16S rRNA gene V3-V4 amplicon PCR from uterine cytobrush samples

For the establishment of a 16S rRNA gene V3-V4 amplicon PCR protocol for equine uterine cytobrush samples, different PCR kits and amplification conditions were tested (data not shown). Most of the PCR kits tested did not specify to be free of bacterial DNA, and with the high number of PCR cycles (35) needed for sufficient amplification of the V3-V4 amplicon, a weak product in the non-template control (DNA-free water) was obtained for these kits (data not shown). Therefore, the Platinum™ Taq DNA Polymerase, DNA-free, was selected for 16S V3-V4 amplicon generation, since it did not reveal a product in the non-template control (Fig. S1).

**Fig. 1:**
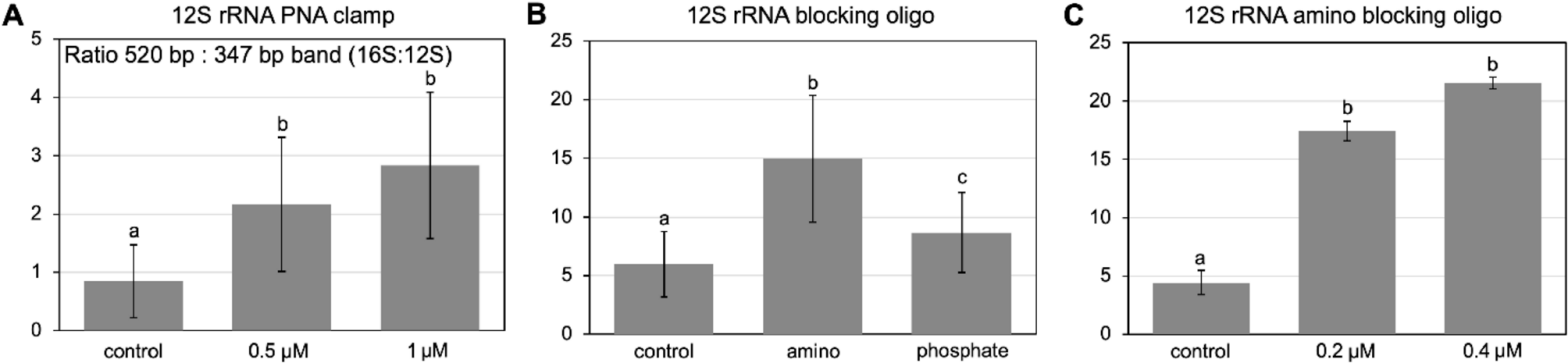
Suppression of the unspecific amplification product derived from the equine mitochondrial 12S rRNA gene. **(A)** 12S rRNA gene peptide nucleic acid (PNA) PCR clamp at concentrations of 0.5 and 1 µM. **(B)** Oligonucleotide blocking the Pro805R primer binding site in the equine mitochondrial 12S rRNA gene with 3’-amino or -phosphate modification (0.2 µM final concentration). **(C)** 12 S rRNA gene blocking oligonucleotide with 3’-amino modification at a final concentration of 0.2 and 0.4 µM. Imaging and quantification were performed with the ChemiDoc MP system (302 nm UV transilluminator) and ImageLab software v.6.1. (Bio-Rad Laboratories). The ratio between the 16S rRNA V3-V4 amplicon product and the unspecific amplification product was calculated from background-subtracted band intensities. Different letters indicate significant differences (Student’s *t*-test).

The PCR for amplification of the 16S rRNA gene V3-V4 region (starting from DNA) based on the original publication for the Pro341F and Pro805R primers^21^ resulted in a significant unspecific amplification product with a size of approximately 350 bp in addition to the expected V3-V4 product (Fig. S1 A). Sequencing of the isolated band revealed that the product was derived from the equine mitochondrial 12 S rRNA gene (Fig. S1 B, C). To suppress this unspecific product, two approaches were applied, a PNA PCR clamp targeting the amplified region of the equine 12 S rRNA gene and an oligonucleotide (12 S rRNA blocking oligo), blocking the binding site of the Pro805R primer in the equine 12 S rRNA gene, but not interfering with amplification of the 16S V3-V4 region (Fig. S1-C). The PNA and 12 S rRNA blocking oligo sequences were designed to be specific to the 12 S rRNA targeting regions not similar between 16S and 12 S rRNA. Figure 1 shows the quantification of the ratios between the 520 bp 16S and the 347 bp 12 S bands from agarose gels for all the approaches tested in comparison to controls without PNA or 12 S rRNA blocking oligo.

For the PNA PCR clamp, the ratios between the 520 bp 16S and the 347 bp 12 S bands for 0.5 µM and 1 µM (final concentration) are shown in Fig. 1A (agarose gel images in Fig. S2 A). Both concentrations showed a significant increase (Student’s *t*-test, *p* = 0.028 and 0.029, respectively) of the ratio (2-3-fold) compared to the control without a significant difference between 0.5 µM and 1 µM PNA clamp (*p* = 0.261).

For the 12S blocking oligonucleotide, both 3’-amino and 3’-phosphate modified oligonucleotides reduced the amplification of the unspecific product (*p* = 0.011 and *p* = 0.015, respectively) (Figs. 1B and S2B), but with higher efficiency for the 3’-amino oligonucleotide (*p* = 0.015). The 0.4 µM concentration of the 3’-amino 12 S blocking oligonucleotide showed almost complete suppression of the 347 bp 12 S rRNA gene amplification product (Fig. 1C, Fig. S2 C). Among the approaches tested, the 3’-amino oligonucleotide was most efficient and was further used for both DNA- and RNA-based amplicon generation protocols.

Next, an additional slight modification of the amplicon PCR by adding Pro341F and Pro805R primers without Illumina adaptor sequences in low concentration was performed to further increase sensitivity. Then, the established 16S rRNA gene V3-V4 amplicon PCR protocol was tested with a serial dilution of the ZymoBIOMICS Microbial Community DNA Standard from 10 pg to 0.15625 pg (performed in triplicate). The amplification of the 16S V3-V4 product was still detectable in all three replicates for the highest dilution of 0.15625 pg (Fig. S3). Considering the average genome size of the bacteria represented in the standard (3.868 Mb), 0.15625 pg of the standard corresponds to approximately 38 bacterial genome copies (= 38 bacterial cells).

For the RNA-based 16S rRNA gene V3-V4 amplicon PCR protocol, the unspecific 12S rRNA product was suppressed in the same way with the 3’-amino 12 S blocking oligo during amplicon PCR and additionally, during cDNA synthesis which was primed with the Pro_805R primer (data not shown). Since the PCR for RNA-based amplicon generation was performed with two cycles less and product yield was overall higher compared to the corresponding DNA-based amplicon (compare Fig. S4 A and S4 B), an up to ten-fold higher sensitivity could be estimated.

In addition to the above-mentioned control for potential bacterial DNA contaminations in reagents used for PCR, additional controls were performed for the elution buffers of the RNA/DNA isolation kit (buffers EB and water), as well as for a fresh cytobrush dropped into the RLT Plus lysis buffer followed by isolation of RNA and DNA for this control (control for contaminations derived from cytobrush and RNA/DNA isolation kit). The results, i.e., no detectable contaminations are shown in Figure S5.

### Comparison of RNA- and DNA-based 16S rRNA gene V3-V4 amplicon sequencing results

DNA and RNA were isolated from 14 uterine cytobrush samples collected from 14 mares, obtaining paired RNA and DNA samples from each uterine sample with a total of 28 samples. Most of the mares were diagnosed as subfertile due to persistent breeding-induced endometritis (PBIE) or without clinical indications but several unsuccessful breeding attempts (Table S2). The obtained nucleic acid concentrations ranged from 11.5 to 87.5 ng/µl for DNA and 22.8-406.8 ng/µl for RNA. For the RNA samples, the RNA integrity number (RIN) was from 8.4 to 10 (median = 9.55). The 16S rRNA gene V3-V4 amplicons were generated for the 28 samples and isolated from agarose gels. Ten selected paired samples are shown in Fig. S4-A for DNA and S4-B for RNA. The concentrations of the obtained amplicon products ranged between 0.5 and 27 ng/µl (median: 2.8) for the DNA-based samples and 3.3–28 ng/µl (median: 13.5) for the RNA-based samples.

Illumina sequencing revealed a total of 62.5 million raw paired-end reads (1.6-3.0 million per sample), and 44.5 million reads (1.1–2.2 million reads per sample) remained after quality trimming/filtering and combining reads 1 and 2. In total, 23,471 amplicon sequence variants (ASVs) were obtained for sequences from all samples, with 8,386 for DNA and 16,907 for RNA samples. For the individual samples, read counts were > 0 for 668 to 4,688 ASVs when ASVs were generated based on all samples (Table S3).

**Fig. 2:**
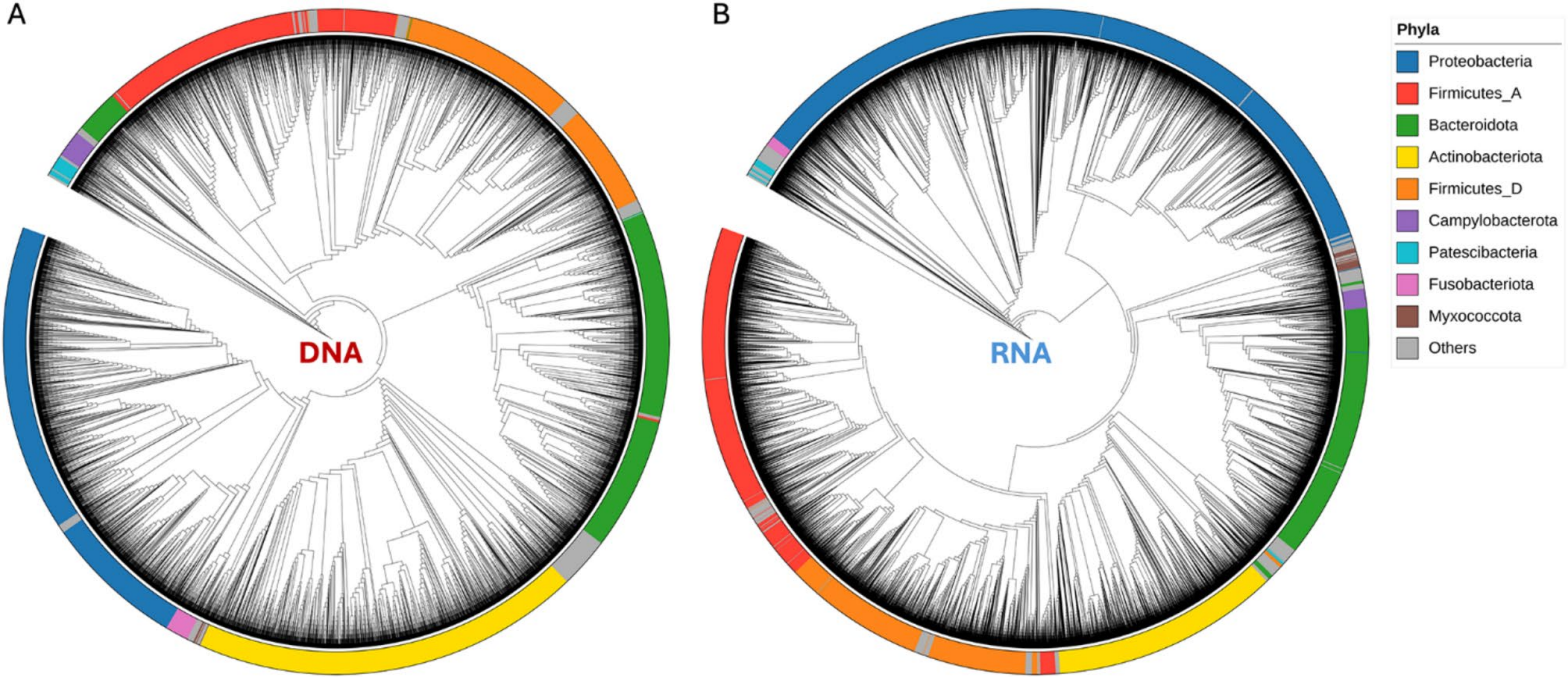
Phylogenetic tree reconstruction of all ASVs for DNA- (A) and RNA-based (B) analysis. Phylogenetic trees for DNA and RNA samples were constructed with FastTree^28^, and the rooted trees were generated using iTOL^29^. The nine most abundant phyla are indicated in different colors in the outer ring.

Both phylogenetic trees constructed from the ASVs detected for DNA-based and RNA-based sequencing revealed a diverse community structure shown in Fig. 2A and B, respectively. The five most abundant phyla (Proteobacteria, Firmicutes_A, Bacteroidota, Actinobacteriota, and Firmicutes_D) showed similar representation for DNA-based and RNA-based analysis (Fig. 2). The comparison of ASVs obtained for RNA- and DNA-based 16S analysis after assignment to taxonomy at level 7 (species), identified 716 (41%) species in common between RNA- and DNA-based analysis. The RNA-based analysis identified 909 species not found with the DNA-based analysis and DNA-based sequencing identified 106 species not present for RNA-based analysis (see Table S4).

**Fig. 3:**
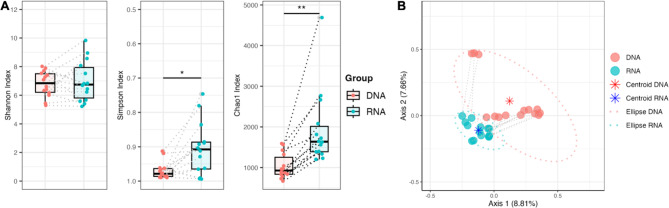
Comparison of alpha- and beta-diversity between DNA- and RNA-based 16S rRNA amplicon sequencing results. **(A)** Comparison of alpha-diversity between DNA- and RNA-based analysis for Shannon, Simpson, and Chao1 indices. **(B)** Principal coordinate analysis (PCoA) illustrating beta diversity of DNA and RNA samples was performed using Jaccard distance. Centroids and ellipses were calculated to depict location and dispersion. Each sample is represented by a dot and dotted lines connect paired samples.

Alpha diversity analysis was performed for different indices and compared between DNA- and RNA-based analysis. No significant difference was found for Shannon index (*p* > 0.588; t-test paired data) (Fig. 3A). Significant differences were observed for Simpson and Chao1 indices (*p* < 0.007 and *p* < 0.0001, respectively; Wilcoxon test paired data) (Fig. 3A). Beta diversity using Principal Coordinate Analysis (PCoA) with Jaccard distance revealed a distinct separation of the DNA- and RNA-based samples (Fig. 3B). DNA-based samples were more diverse for Jaccard distance compared to RNA-based samples. Permutational analysis of variance (PERMANOVA, Bray-Curtis dissimilarity) analysis also revealed a significant difference between DNA- and RNA-based samples (*p* < 0.001).

**Fig. 4:**
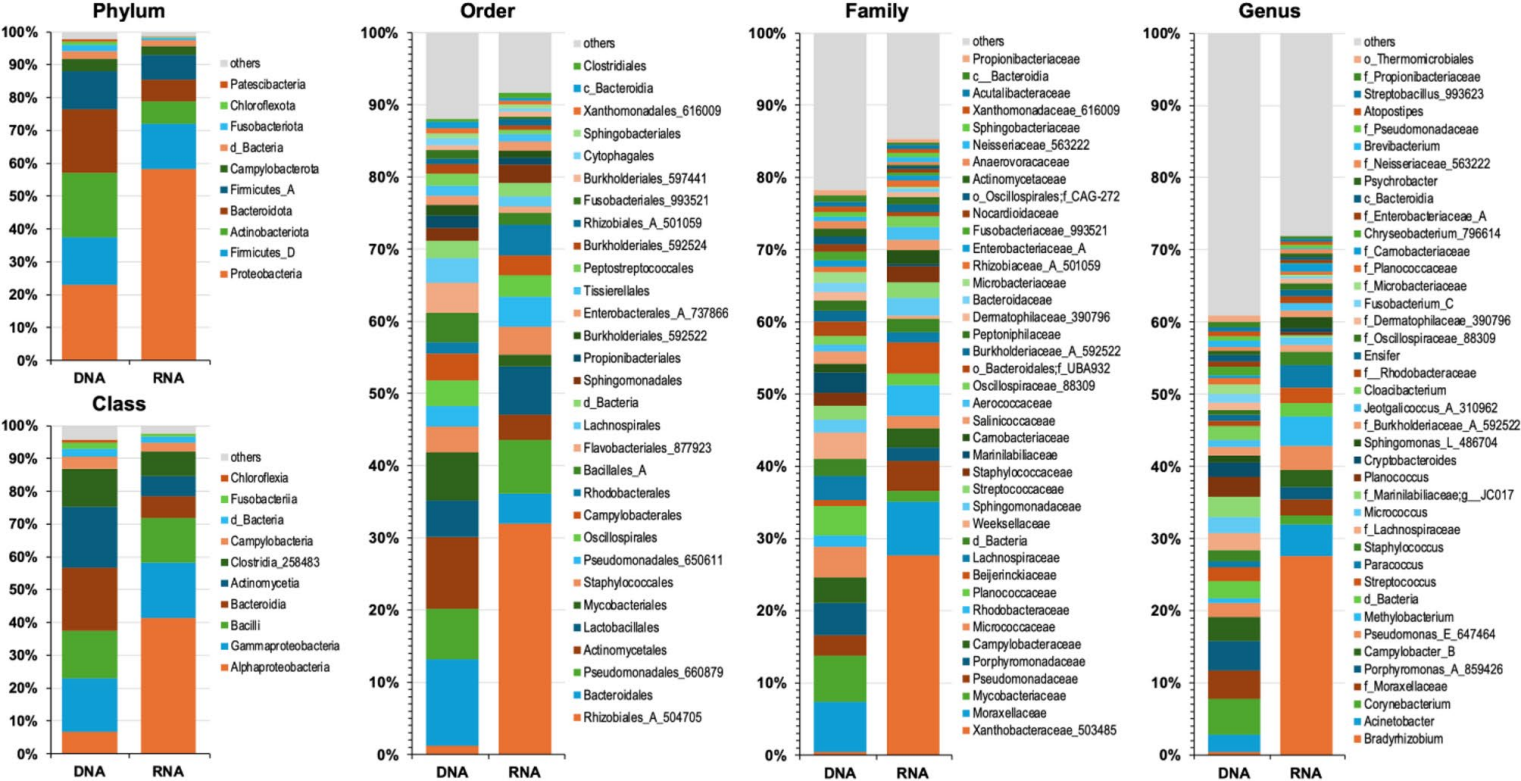
Taxonomic composition for DNA- and RNA-based 16S rRNA analysis. The taxonomy bar plots show the average taxonomic composition of DNA and RNA samples for the taxonomic levels: phylum, class, order, family, and genus.

The taxonomic composition for grouped DNA and RNA samples is shown in Fig. 4 for taxonomic levels 2 (phylum) to 6 (genus). At the phylum level, Proteobacteria, Firmicutes_D, Actinobacteriota, Bacteroidota, and Firmicutes_A were the predominant taxa for both DNA- and RNA-based analysis. *Proteobacteria* had the highest relative abundance for both DNA and RNA samples, being much higher for RNA samples (Fig. 4, top left). The relative abundance of Firmicutes_D was similar. Actinobacteriota, Bacteroidota, and Firmicutes_A showed higher relative abundance for DNA-based analysis (see also Table S4). Overall, the taxonomic composition was similar between DNA- and RNA-based analysis at the levels 3–5 (class to family) with the main difference of the high overrepresentation of *Bradyrhizobium* and the corresponding phyla at the higher taxonomic levels (Fig. 4 and Table S4). In addition, the RNA-based analysis revealed higher diversity for taxa with very low relative abundance (Table S4).

**Fig. 5:**
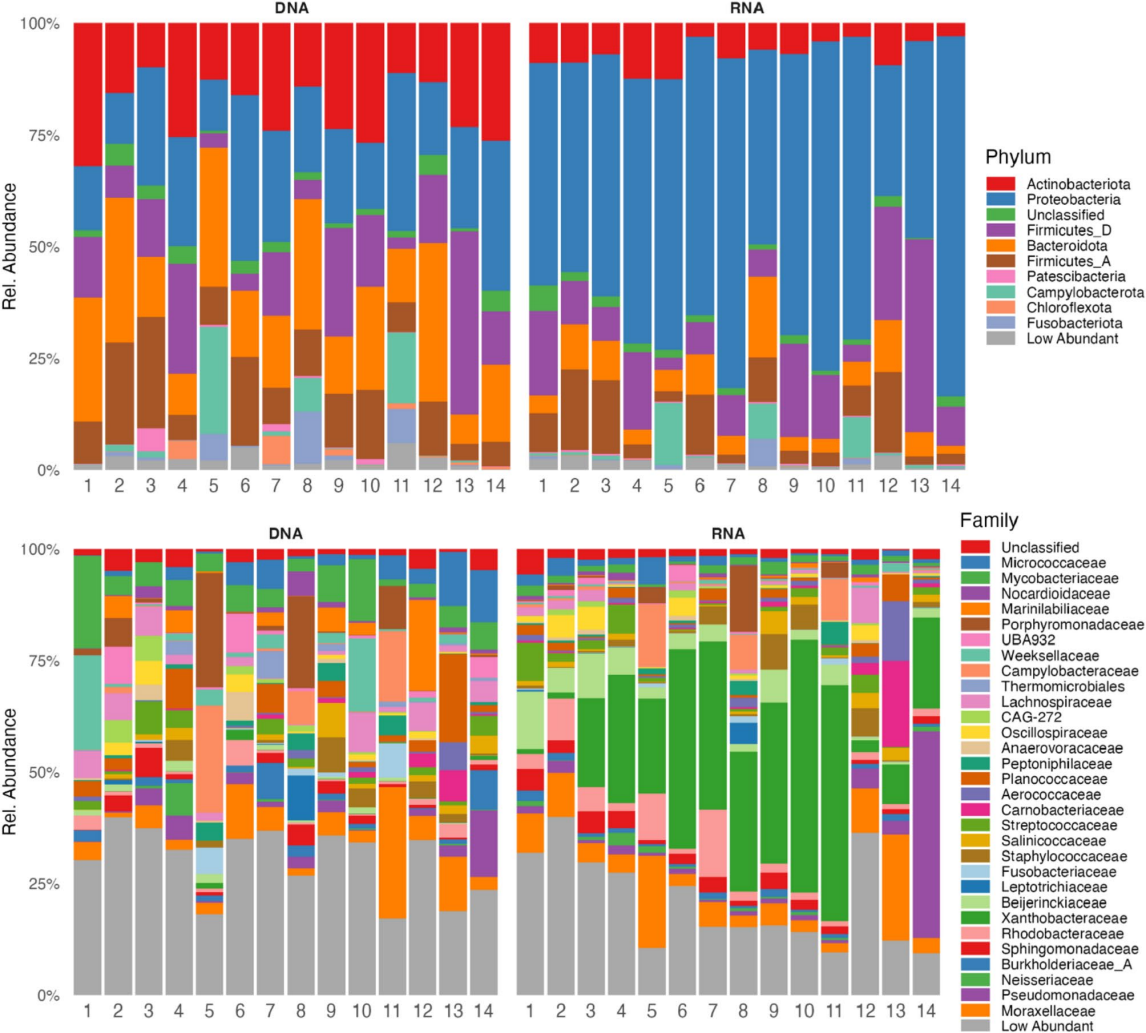
Taxonomic composition of individual DNA and RNA samples. Taxa with a relative abundance of > 5% in at least one sample are shown (10 most abundant for phylum and 31 most abundant taxa for family).

The taxonomic composition of the individual paired DNA- and RNA-based samples at different phylogenetic levels is shown in Fig. 5 (phylum and family levels) and Fig. S6 (class, order, and genus levels, see also Table S4). Mainly at the more specific levels, the comparison of the different cytobrush samples (different mares) showed similarities in their taxonomic composition but also some distinct individual differences. Some mares showed clear differences from the other mares, e.g., samples 1, 5, 10–14 (Figs. 5 and S5). The DNA- and RNA-based analysis of the same sample showed overall similar patterns (Figs. 5 and S5). Samples 1 and 10–13 showed differences between DNA- and RNA-based analysis with some taxa predominantly present for the DNA-based analysis.

**Fig. 6:**
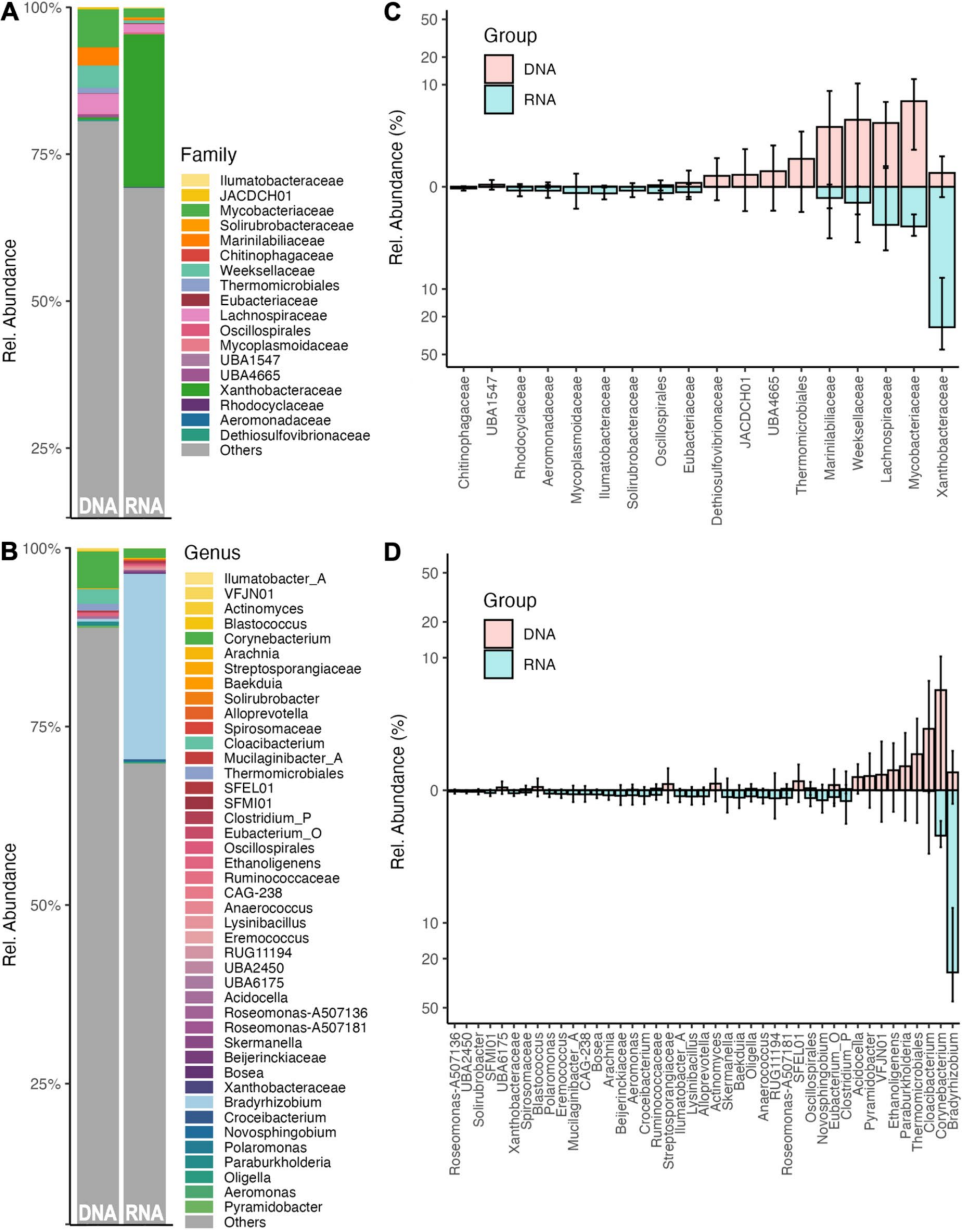
Identification of differentially abundant taxa between DNA- and RNA-based 16S rRNA analysis. **(A)** and **(B)** Taxonomy bar plots showing differentially abundant taxa between DNA and RNA samples at family (level 4) and genus (level 6) level, respectively. **(C)** and **(D)** Box plots showing the relative abundance of differentially abundant taxa between DNA and RNA samples for the family and the genus level, respectively. Y-axis is in logarithmic scale (log 10).

The differential analysis of microbiome composition between RNA- and DNA-based analysis revealed significant differences at different taxonomic levels (family and genus shown in Fig. 6, other levels in Fig. S7, all in Table S5). The genera with the highest differences (top 5 higher for DNA and top 10 higher for RNA) are shown in Table 2. For the phyla with high relative abundance, more phyla with increased relative abundance in the DNA-based analysis compared to RNA-based analysis were obtained (Fig. 6C, D). A few taxa with high relative abundance and increased for RNA- vs. DNA-based analysis were also found, e.g., the *Rhizobiales_A_504705 families Xanthobacteraceae* and *Beijerinckiaceae*. In contrast, most of the differential phyla with low relative abundance were much higher for RNA-based analysis, and some of them were only detected with the RNA-based analysis.

**Table 2:** Genera with the highest log2 fold changes RNA- versus DNA-based 16S rRNA V3-V4 amplicon sequencing analysis (top 5 genera higher for DNA-based analysis and top 10 genera higher for RNA-based analysis). *mean of pairwise log2 fold changes of RNA- vs. DNA-based relative abundance.

	Mean relative abundance					
Genus or higher taxonomic level	All	DNA	RNA	log2 FC RNA/DNA*	Wilcoxon Rank test p-value	Adj. p-value
*Acidocella*	0.135%	0.269%	< 0.001%	-5.71	0.0000	0.0055
*Thermomicrobiales*	0.464%	0.924%	0.003%	-5.61	0.0000	0.0005
*Paraburkholderia_580243*	0.272%	0.543%	< 0.001%	-5.13	0.0000	0.0002
*Cloacibacterium*	1.027%	2.040%	0.014%	-4.56	0.0011	0.0481
*Ethanoligenens*	0.218%	0.436%	0.001%	-3.94	0.0012	0.0451
*Bosea*	0.041%	0.004%	0.078%	4.89	0.0006	0.0253
*Alloprevotella*	0.061%	0.007%	0.116%	4.92	0.0004	0.0208
*Arachnia*	0.048%	0.001%	0.094%	5.13	0.0003	0.0163
*Baekduia*	0.071%	< 0.001%	0.142%	5.20	0.0001	0.0080
*Croceibacterium_484881*	0.056%	< 0.001%	0.111%	5.32	0.0001	0.0072
*Novosphingobium_485351*	0.101%	0.004%	0.197%	5.50	0.0000	0.0018
*Ilumatobacter_A*	0.060%	0.006%	0.113%	5.50	0.0002	0.0114
*Roseomonas_A_507181*	0.087%	0.028%	0.145%	5.67	0.0001	0.0085
*Anaerococcus*	0.072%	0.007%	0.137%	6.15	0.0000	0.0025
*Bradyrhizobium*	13.157%	0.382%	25.932%	6.84	0.0000	0.0020

To find differences between DNA- and RNA-based analysis indicating the presence of dead bacteria or free bacterial DNA in a sample, the relative abundance of the DNA- and RNA-based analysis was compared for the paired samples (based on Table S4, 6-Genus). Taxa were only considered if the relative abundance for the DNA sample was at least 1%. Many taxa were found with much higher relative abundance for DNA samples. Taxa with a difference of more than 100-fold are listed in Table 3. The five taxa with the highest difference were Paraburkholderia_580243, Cloacibacterium, Chitinophagales, Peptostreptococcaceae_256921, and Ethanoligenens.

**Table 3:** Taxa with at least 100-fold higher relative abundance for DNA- compared to RNA-based analysis of the same sample and > 1% relative abundance for the DNA-based analysis.

Genus or higher taxonomic level(s)	FC DNA vs. RNA	rel. Ab. DNA	Sample ID
*Paraburkholderia_580243*	14,482	4.9%	E4_E_12
*Cloacibacterium*	10,248	10.6%	E4_U_03
*o_Chitinophagales*	5656	1.0%	E4_F_01
*f_Peptostreptococcaceae_256921*	3587	1.5%	E4_E_12
*Ethanoligenens*	3484	2.6%	E1_ES_02
*f_Eggerthellaceae*	2217	2.4%	E1_ES_09
*o_Thermomicrobiales*	1665	6.1%	E4_E_12
*o_Acidimicrobiales; f_JACDCH01;g_VFJN01*	1413	4.1%	E4_E_12
*Lawsonella*	1387	2.3%	E4_E_12
*Sphingomicrobium_483265*	1137	1.9%	E4_F_01
*Peptoniphilus_E*	655	1.4%	E4_U_05
*Dyadobacter_906144*	615	1.6%	E1_ES_02
*f_Burkholderiaceae_A_595422*	579	1.5%	E4_F_01
*Marmoricola_A_392027*	489	2.2%	E4_E_03
*f_Chitinophagaceae_966727*	320	4.8%	E4_U_17
*Microthrix*	276	1.1%	E4_E_05
*f_Cellulomonadaceae*	275	1.6%	E1_ES_02
*f_Microbacteriaceae*	244	2.6%	E4_E_09
*Quadrisphaera*	151	1.1%	E4_E_09
*Spirosoma*	105	2.6%	E4_U_17

## Discussion

The main purpose of this study was to compare the RNA- and DNA-based 16S rRNA amplicon sequencing approaches for the analysis of the equine uterine microbiome. Given the low bacterial biomass of the uterus, particular attention was given to the sensitivity of microbiota detection and differences in the composition and diversity of the microbiome provided by both approaches. This included the establishment of a sensitive and specific 16S V3-V4 amplicon generation protocol from nucleic acids isolated from uterine cytobrush samples to perform a robust and reproducible microbiota analysis of the low biomass uterine microbiome^34^.

One critical problem of the high sensitivity of PCR protocols established for very low-biomass microbiomes is the amplification of even tiny amounts of contaminating bacterial DNA, e.g., contained in most PCR reagents not certified as DNA-free or derived from other sources during sample collection and processing^35,36^. Such contaminations can critically affect the results and lead to an overestimation of diversity and misinterpretation of community composition^35,36^. The use of PCR kits certified as free of bacterial DNA did not show any amplicon product in the non-template control. Furthermore, we did not find detectable contaminations in further controls including a “mock cytobrush”, i.e., a fresh cytobrush dipped into the lysis buffer of the RNA/DNA isolation kit with subsequent isolation of RNA and DNA as performed for the real samples. However, it is difficult to exclude minimal contaminations when analyzing low-biomass microbiomes. This should not be critical for comparative uterine microbiome studies where such minimal contaminations could be in samples of all experimental groups. In the case of the present study, we tried to exclude potential contaminations with very low abundance using a filter to remove ASVs with very low frequencies. Furthermore, we avoided to discuss taxa which were differential between DNA- and RNA-based approaches but were only found with very low read counts.

Another problem of the analysis of low biomass microbiomes and the presence of high amounts of host material in the sample at the same time can be the amplification of homologues sequences from host ribosomal RNAs^37–39^. Despite only partial similarities of the 16S V3-V4 primers, the co-amplification of a 12S rRNA fragment was observed probably due to the very high ratio of host to bacterial DNA in the uterine cytobrush samples. Based on studies using peptide nucleic acid (PNA) PCR clamps to suppress amplification of host rRNA sequences^37,38,40^, a PNA targeting the amplified fragment of the equine mitochondrial 12S rRNA gene was designed and tested. Since the 12S PNA clamp did not reveal a complete suppression of the unspecific product, an oligonucleotide was designed to compete with binding of the Pro805R primer in the 12S rRNA sequence. A specific blocking of the binding site in the 12S rRNA was accomplished by designing the oligonucleotide sequence to overlap the Pro805R binding site with the 5’ half while the 3’ half was only complementary to the subsequent 12 S rRNA-specific region. This approach showed higher efficiency and was further used in the established DNA- and RNA-based amplicon generation protocol.

Although, this established protocol was very specific, the sensitivity was not high enough to obtain sufficient amounts of V3-V4 amplicon product for all cytobrush DNA samples for Illumina library preparation. To avoid the introduction of additional amplification bias by adding more cycles or performing two rounds of amplification (also resulting in a higher number of cycles) in a nested or semi-nested PCR^34^, V3-V4 primers without Illumina adaptor sequences were added to the reaction. Due to the advantage of shorter oligonucleotides with respect to diffusion and hybridization kinetics^41^, the idea was that the V3-V4 primers without Illumina adaptor sequences are primarily used during the initial PCR cycles and thereby increasing PCR efficiency. The V3-V4 primers with Illumina adaptor sequences were predominantly used later during the PCR, thereby incorporating the adaptor sequences necessary for library construction. Indeed, with this modification, the amplicon generation was successful for all 14 tested cytobrush DNA samples and enabled a detection sensitivity comparable to previous studies using semi-nested^34^ or droplet digital PCR^42^. As a comparison regarding detected genome copy numbers, a previously published 16S rRNA gene PCR to detect contaminant mycoplasmas in cell cultures^43^ detected 10 copies of mollicute target DNA, but used specific 16S primers for Mollicutes species and 40 cycles of PCR, indicating the high sensitivity of the presented 16S rRNA V3-V4 amplicon PCR protocol.

The results of the 16S rRNA V3-V4 amplicon PCR in comparison of the RNA-based and DNA-based approach already showed a higher sensitivity for the RNA-based amplicon generation pointing at a higher 16S rRNA target concentration in the input sample for the amplicon PCR. The main advantage of the RNA-based 16S amplicon generation is probably the much higher copy number of the 16S rRNA (= number of ribosomes per cell, e.g. 20,000–30,000 for *E. coli*)^18^ compared to the 16S rRNA gene copy number (1–15 per genome/cell)^15^, resulting in a much higher concentration of 16S amplicon PCR target molecules in the sample for the RNA-based approach.

Although, some studies raised concerns about additional bias or errors introduced by the reverse transcription of 16S rRNA into cDNA^44^, others showed a rather low error rate for typical M-MuLV RT enzymes (less than 1 × 10^–4^/bp)^45^, which is in the range of *Taq* polymerase (4.3 × 10^− 5^/bp)^46^. Due to the lower number of PCR cycles for RNA-based amplicon PCR, the error rates should be comparable between the DNA- and RNA-based approach. To avoid potential bias regarding synthesis errors and processivity, an engineered M-MuLV RT was used, which is highly sensitive, efficient, and thermostable. Furthermore, cDNA synthesis was primed with the Pro805R primer, thereby restricting as much as possible cDNA synthesis to the 16S rRNA.

Regarding the part of the study comparing the microbiome composition between DNA- and RNA-based analysis using 14 cytobrush samples collected from 14 different mares, a similar species diversity based on the Shannon alpha diversity index^47^ was found. But this index combines richness and evenness, i.e., measures both the number of species and the inequality between species abundances. The RNA-based analysis detected a much higher number of ASVs, but the evenness was lower compared to the DNA-based analysis resulting in a similar Shannon index. Consequently, the significant differences in Simpson^48^ and Chao1 ^49^ indices reflected the higher “species richness” for the RNA-based analysis, which was probably mainly based on the higher sensitivity of the RNA-based approach for detecting taxa with lower abundance. The significant differences in beta diversity (Bray-Curtis dissimilarity and Jaccard distance) indicated differences in microbial abundances between both approaches and differences for the presence or absence of species, respectively. The latter was probably again due to the higher sensitivity of the RNA-based approach, but also due to taxa only found by the DNA-based approach (see below).

The differential microbiome composition analysis between the DNA- and RNA-based approach was expected to show differentially abundant taxa based on (i) the higher sensitivity of the RNA-based approach, (ii) the differences due to rRNA gene copy numbers and numbers of ribosomes, and (iii) the detection of free bacterial DNA or dead bacteria (in which the rRNA is degraded) by the DNA-based approach.

Indeed, the majority of differential taxa with low relative abundance had increased relative abundance for RNA-based samples or were even not detected in DNA-based samples, indicating the higher sensitivity of RNA-based amplicon PCR. In contrast, more of the differential taxa with high relative abundance were increased for DNA-based samples. One reason for this finding could be that the RNA-based approach identified double numbers of ASVs thereby reducing the relative abundance of the taxa with high abundance. On the other side, the dominance of the genus *Bradyrhizobium* in most of the RNA-based samples resulted in a relative decrease in the relative abundance of other taxa in the RNA-based analysis. However, a closer look at taxa with higher relative frequency for DNA- compared to RNA-based analysis revealed for some cytobrush samples a difference of up to 10,000-fold, which suggests the presence of dead bacteria or free bacterial DNA. For example, *Corynebacterium* was found in some samples with a relative frequency for DNA-based analysis of up to 19.4% (13-fold higher than for RNA). In a recent study in mares with pregnancy complications (infertility, endometritis, early pregnancy loss), *Corynebacterium uterequi* was isolated and characterized, and the authors suggested a possible role of *C. uterequi* in the context of the observed pregnancy complications^50^. This indicates that the DNA-based analysis might have detected dead *Corynebacterium* bacteria in some samples while in most samples, DNA- and RNA-based approaches found a similar relative abundance. One of the above-mentioned very high ratios of DNA vs. RNA relative abundance was found for *Cloacibacterium* for some samples which is also suggesting that the DNA-based analysis mainly detected dead *Cloacibacterium*. This bacterium has been found as one of the dominating bacterial species in human endometrium and cervix samples after hysterectomy^51^. Another example for the potential detection of dead bacteria was *Thermomicrobiales*, a typical soil bacterium^52^, which was undetectable or very low in the RNA-based samples, in contrast to DNA-based samples with up to 6% relative abundance. While these findings support the presence of dead bacteria and/or cell-free bacterial DNA, this needs to be validated in future studies, e.g., using the propidium monoazide (PMA) approach^53^ to see if some taxa disappear in the DNA-based analysis with PMA treatment.

The most striking difference between RNA- and DNA-based analysis was found for the relative abundance of *Bradyrhizobium*, which was more than 50% in some RNA samples but less than 1% in most of the DNA samples (sample with the highest difference: 2,856-fold). *Bradyrhizobium* was the most prominent example for inherent bias of RNA- and DNA-based 16S rRNA analysis. DNA-based analysis is mainly biased by the 16S rRNA gene copy number, which can vary from 1 to 15 (21) (https://rrndb.umms.med.umich.edu)^54,55^. *Bradyrhizobium* has only a single rRNA gene copy, resulting in a 5 to 6-fold underrepresentation compared to bacteria with an average copy number. Louca et al. addressed the challenge of correcting for 16S rRNA gene copy numbers in microbiome studies. They found that current methods for predicting gene copy numbers are often inaccurate, leading to biases in community composition estimates^56^. But the copy number alone could not explain the difference between RNA and DNA samples in the case of *Bradyrhizobium*, suggesting an additional reason for the observed difference (on average 62-fold). *Bradyrhizobium* was probably also overrepresented in the RNA samples because these bacteria are relatively big^57^, suggesting that they contain a large number of ribosomes. The example of *Bradyrhizobium* points at the main bias of the RNA-based approach related to the number of ribosomes per cell, which can be from only 1’000 to more than 60’000, depending on cell size and bacterial growth rate^16,19,58^. Several findings from other studies are supporting a functional role for *Bradyrhizobium* in the uterus. *Bradyrhizobium* has been found in the equine placenta under the five most abundant genera^59^ and also in the ovine and human uterus^60,61^.

In addition to cell size/ribosome number and 16S rRNA gene copy numbers, the method of nucleic acid isolation has a great bias on 16S rRNA gene sequencing results^62^. Although, our study isolated DNA and RNA from the same sample lysate, there could be differences in the efficiency of isolating the genomic DNA and the ribosomal RNA. In case of incomplete lysis, it could be possible that ribosomes are released from partially lysed cells but not the genomic DNA. A study comparing different lysis methods revealed higher bacterial diversity and significantly improved DNA extraction efficiency for archaea and some bacteria, including *Clostridium* and *Eubacterium*, using mechanical cell disruption^63^. This could explain for the present study that a much higher relative abundance of *Clostridium* and *Eubacterium* in the RNA-based analysis was found due to incomplete cell disruption, i.e., ribosomal rRNA was efficiently isolated but not the bacterial genomic DNA.

With respect to the equine uterine microbiome and its relationship to uterine receptivity, a combination of both DNA- and RNA-based approaches could offer a complementary perspective on microbiota community composition^64,65^. By amplifying the 16S ribosomal RNA, only present in ribosome-containing bacteria, the RNA-based approach mainly reflects the alive and active bacterial microbiota and could allow conclusions on the functional uterine microbiome at the time of sampling. However, despite bacteria slowing down their ribosome biosynthesis under poor growth conditions^66^, ribosomes can be stored in an inactive form and “hibernate” in dormant bacteria until conditions improve^66,67^. Thus, RNA-based 16S analysis is probably also detecting dormant bacteria in many cases, but at least to a lower extent.

The detection of dead bacteria or cell-free bacterial DNA released from dead bacteria could also be of interest for uterine microbiome analysis, particularly in the context of uterine infections and endometritis, which belong to the main reasons for reduced fertility in the mare^50,68–70^. Dead bacteria could be an indication for a previous infection if the same sample shows for the RNA-based analysis a normal uterine microbiome. As mentioned above, taxa were found with much higher relative abundance for DNA- compared to RNA-based analysis in some of the paired samples. Some of these taxa could be associated with a disturbed uterine microbiome affecting endometrial receptivity, e.g., *Corynebacterium* that has been associated with endometritis and pregnancy complications in the mare^50^. Furthermore, *Moraxellaceae* was found in the bovine uterus in the most abundant taxa^71^ and *Cloacibacterium* in the uterus of women^51^. Moreover, *Micrococcus luteus* infection of the uterus has been related to early embryonic developmental arrest^72^, and *Paraburkholderia* identified in the vagina of women has been associated with recurrent spontaneous abortion^73^. In dairy cows, *Fusobacterium* has been described as one of the uterine pathogens involved in metritis^74^. These examples suggest that a combined DNA- and RNA-based 16S rRNA analysis could bring additional information, e.g., about the active microbiome and recent uterine infections.

## Conclusions

This study established a highly sensitive and specific 16S rRNA V3-V4 amplicon PCR protocol for uterine cytobrush samples. The comparison of DNA- and RNA-based 16S rRNA analysis starting from the same cytobrush sample revealed an overall similar microbiome composition between the two methods. But the results pointed to particular differences, mainly based on the higher sensitivity of the RNA-based approach, the bias due to rRNA gene copy numbers, numbers of ribosomes per cell, and presumably dead bacteria in the samples. The obtained results showed reliability and higher sensitivity of the RNA-based 16S rRNA analysis, while the comparison to the DNA-based approach provided additional relevant information. Altogether, the results indicate a combined DNA- and RNA-based analysis as an approach to obtain complementary and valuable information in the context of fertility-related studies of the uterine microbiome.

## Electronic supplementary material

Below is the link to the electronic supplementary material.

Supplementary Material 1

Supplementary Material 2

Supplementary Material 3

Supplementary Material 4

Supplementary Material 5

Supplementary Material 6

Supplementary Material 7

Supplementary Material 8

Supplementary Material 9

Supplementary Material 10

Supplementary Material 11

Supplementary Material 12

Supplementary Material 13

Supplementary Material 14

## Data Availability

The amplicon sequencing raw data is available at the NCBI’s Sequence Read Archive (SRA) under the BioProject accession ID PRJNA1165023 (http://www.ncbi.nlm.nih.gov/bioproject/1165023). The bioinformatics workflow for data analysis, trained QIIME2 feature classifiers, and other database files are available on ZENODO under project ID 13847011 (https://doi.org/10.5281/zenodo.13847011).
