# Supplementary figures and images for: Comparison of RNA- and DNA-based 16S amplicon sequencing to find the optimal approach for the analysis of the uterine microbiome
Source: Sci Rep. 2025 May 16;15:17037. doi: 10.1038/s41598-025-00969-5 (PMC12084623; doi:10.1038/s41598-025-00969-5)

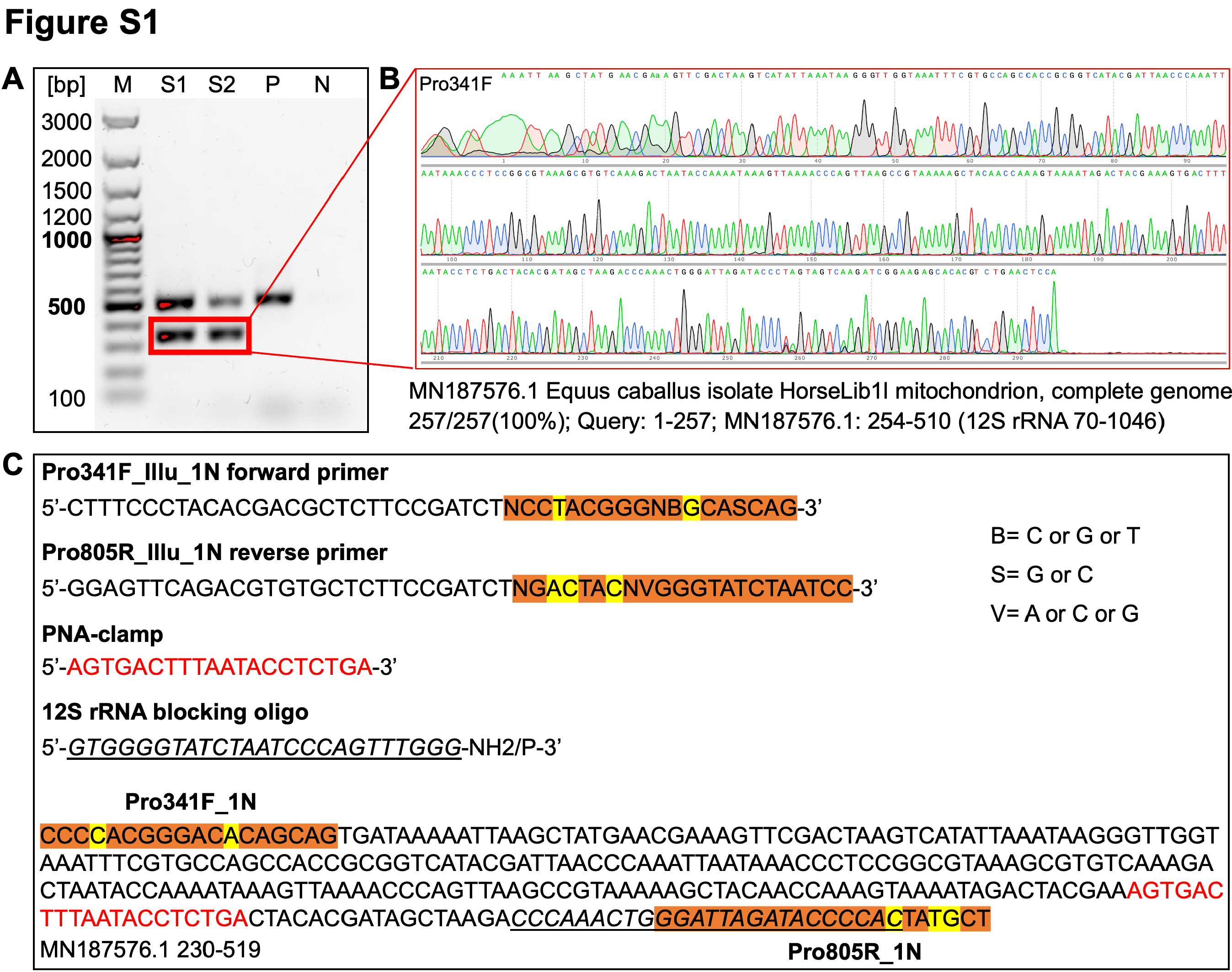

Supplement: Supplementary file 1 — Supplementary Material 1 [file 41598_2025_969_MOESM1_ESM.tif]

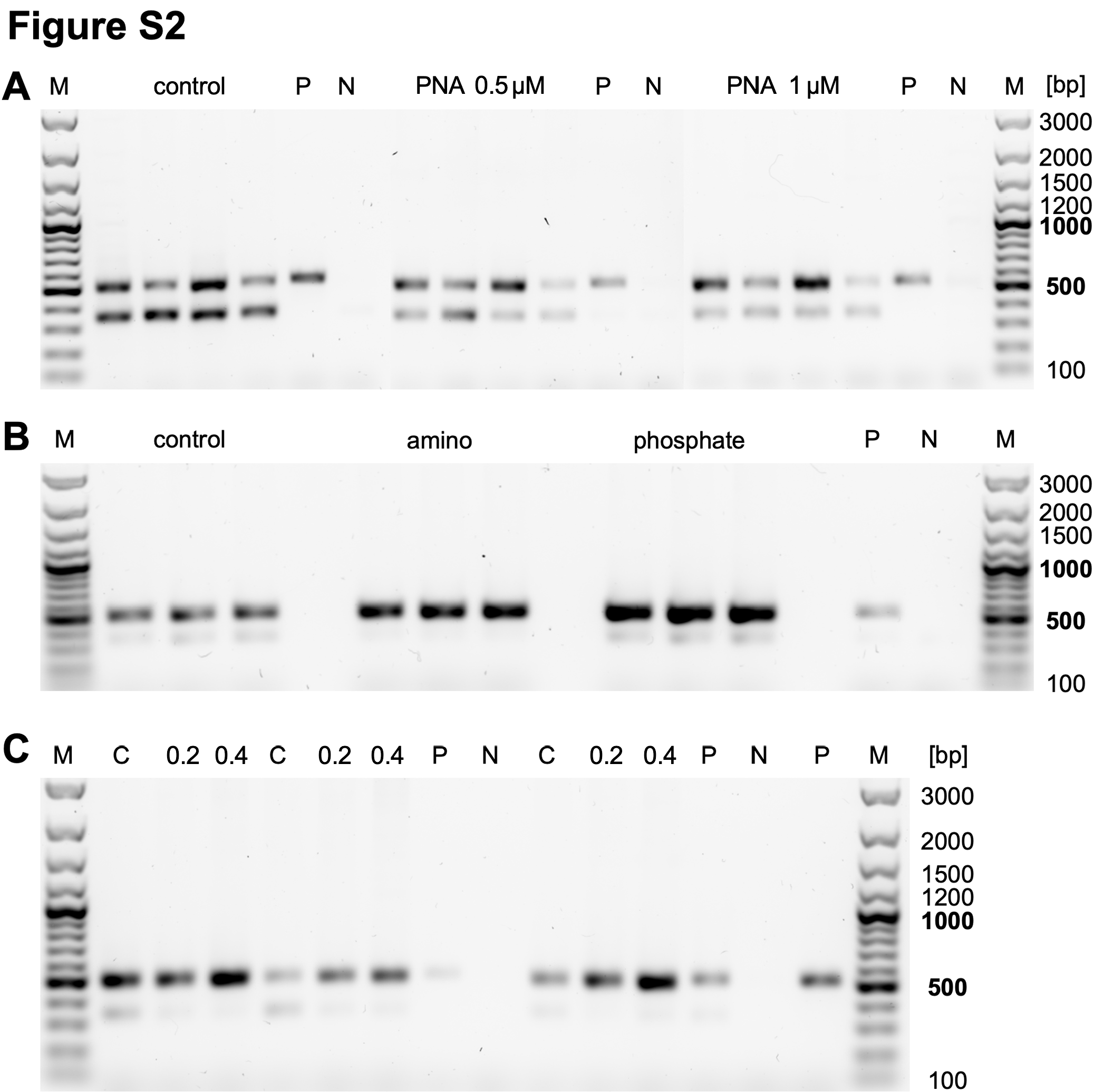

Supplement: Supplementary file 2 — Supplementary Material 2 [file 41598_2025_969_MOESM2_ESM.tif]

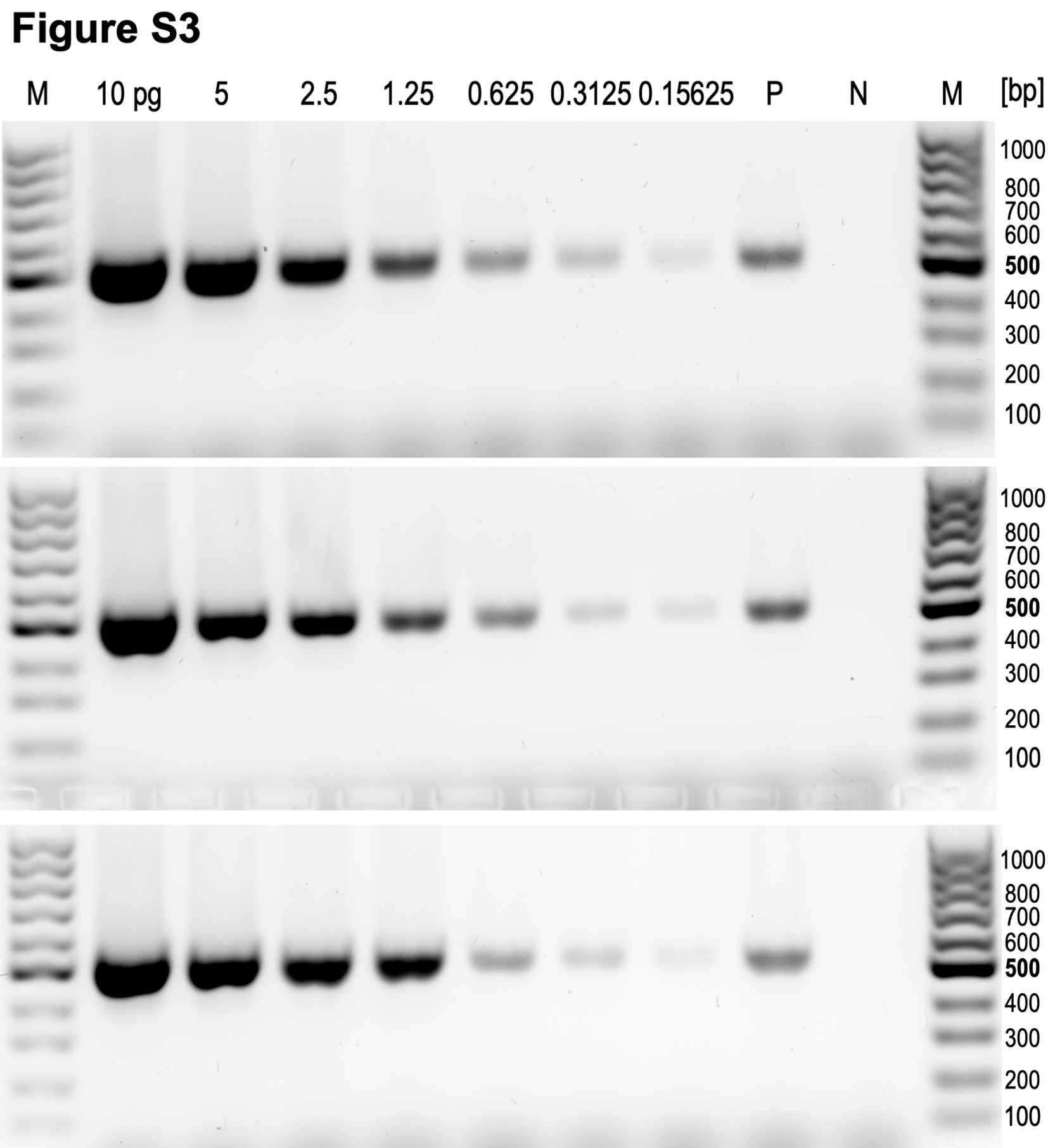

Supplement: Supplementary file 3 — Supplementary Material 3 [file 41598_2025_969_MOESM3_ESM.tif]

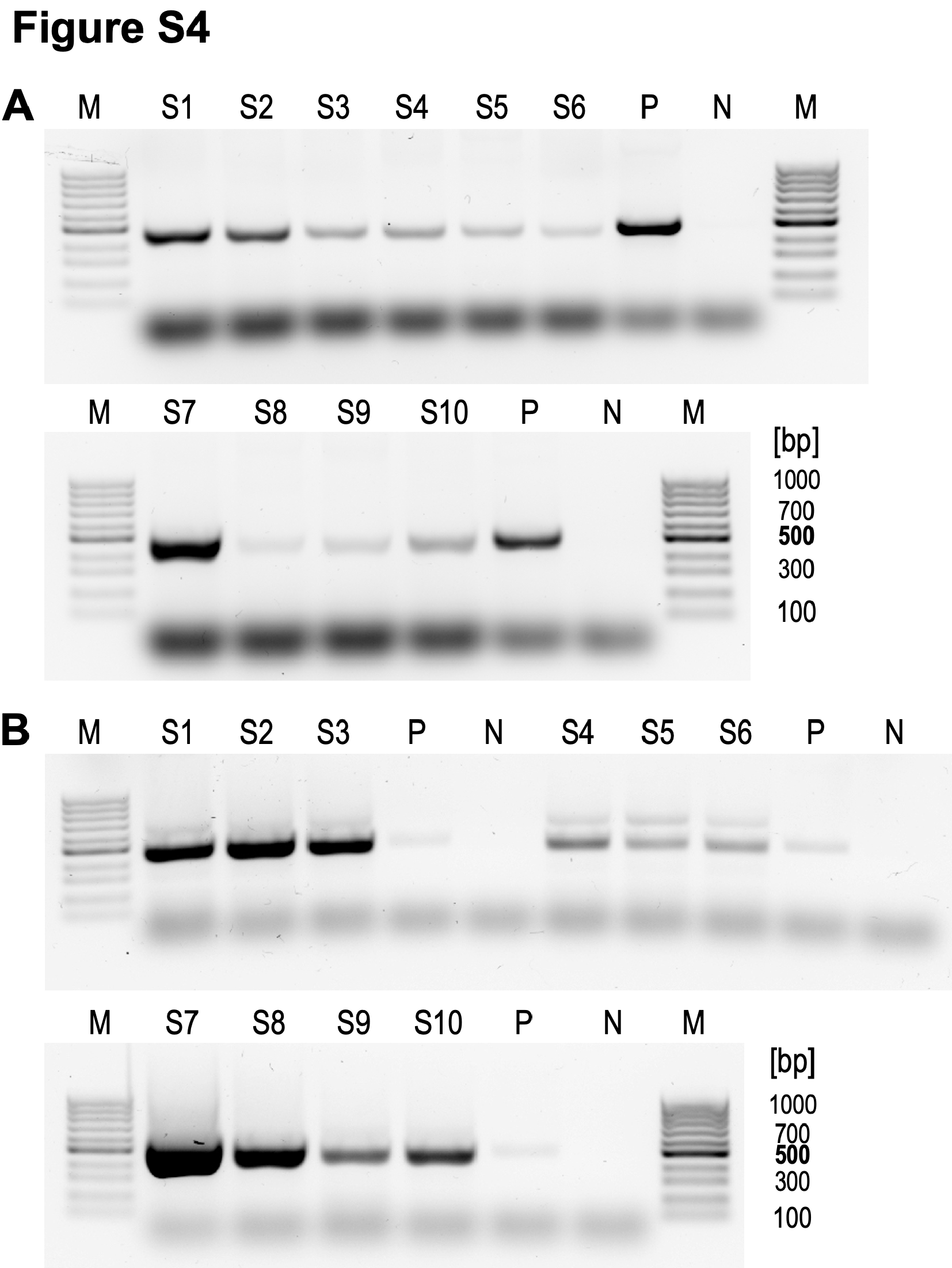

Supplement: Supplementary file 4 — Supplementary Material 4 [file 41598_2025_969_MOESM4_ESM.tif]

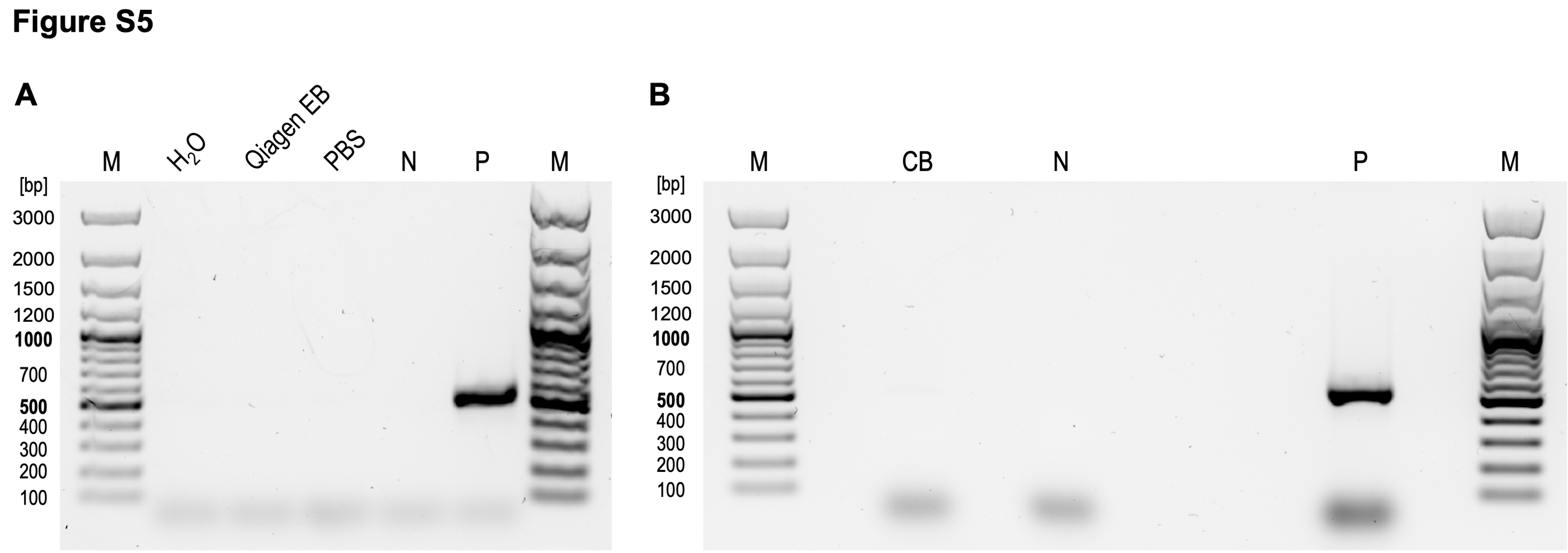

Supplement: Supplementary file 5 — Supplementary Material 5 [file 41598_2025_969_MOESM5_ESM.tif]

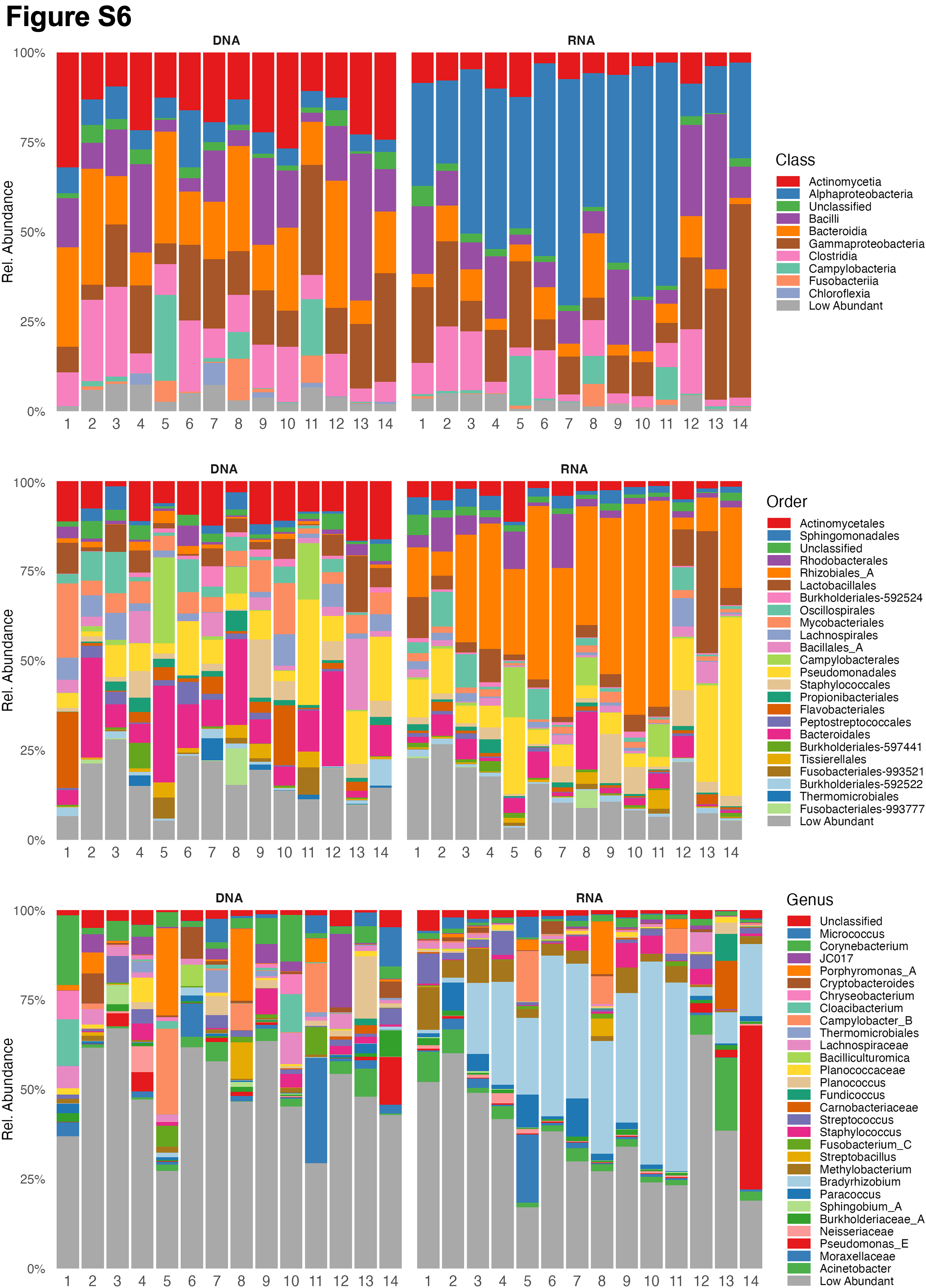

Supplement: Supplementary file 6 — Supplementary Material 6 [file 41598_2025_969_MOESM6_ESM.tif]

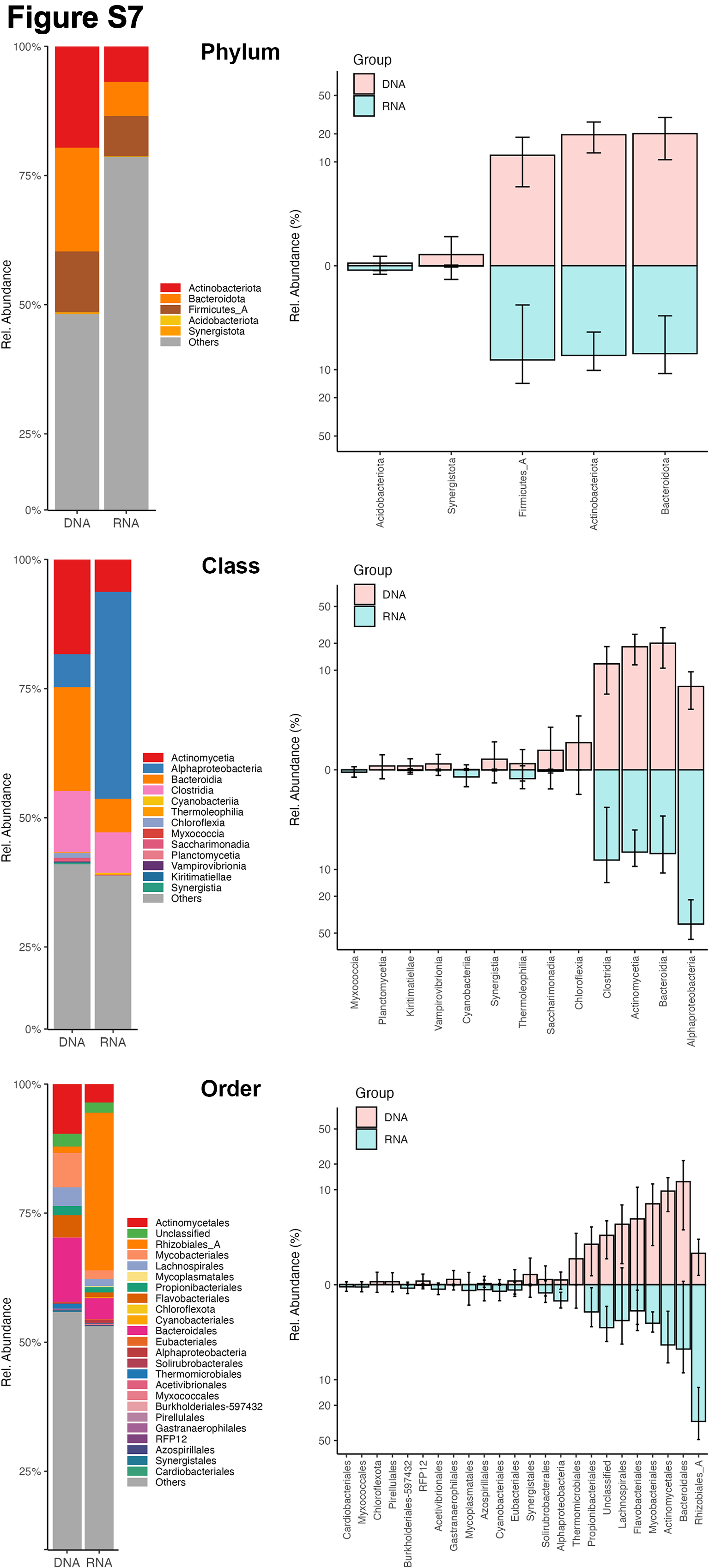

Supplement: Supplementary file 7 — Supplementary Material 7 [file 41598_2025_969_MOESM7_ESM.tif]
