# Supplementary material for: Comparison of RNA- and DNA-based 16S amplicon sequencing to find the optimal approach for the analysis of the uterine microbiome
Source: Sci Rep. 2025 May 16;15:17037. doi: 10.1038/s41598-025-00969-5 (PMC12084623; doi:10.1038/s41598-025-00969-5)

Full images of agarose gel electrophoresis

Figure S1

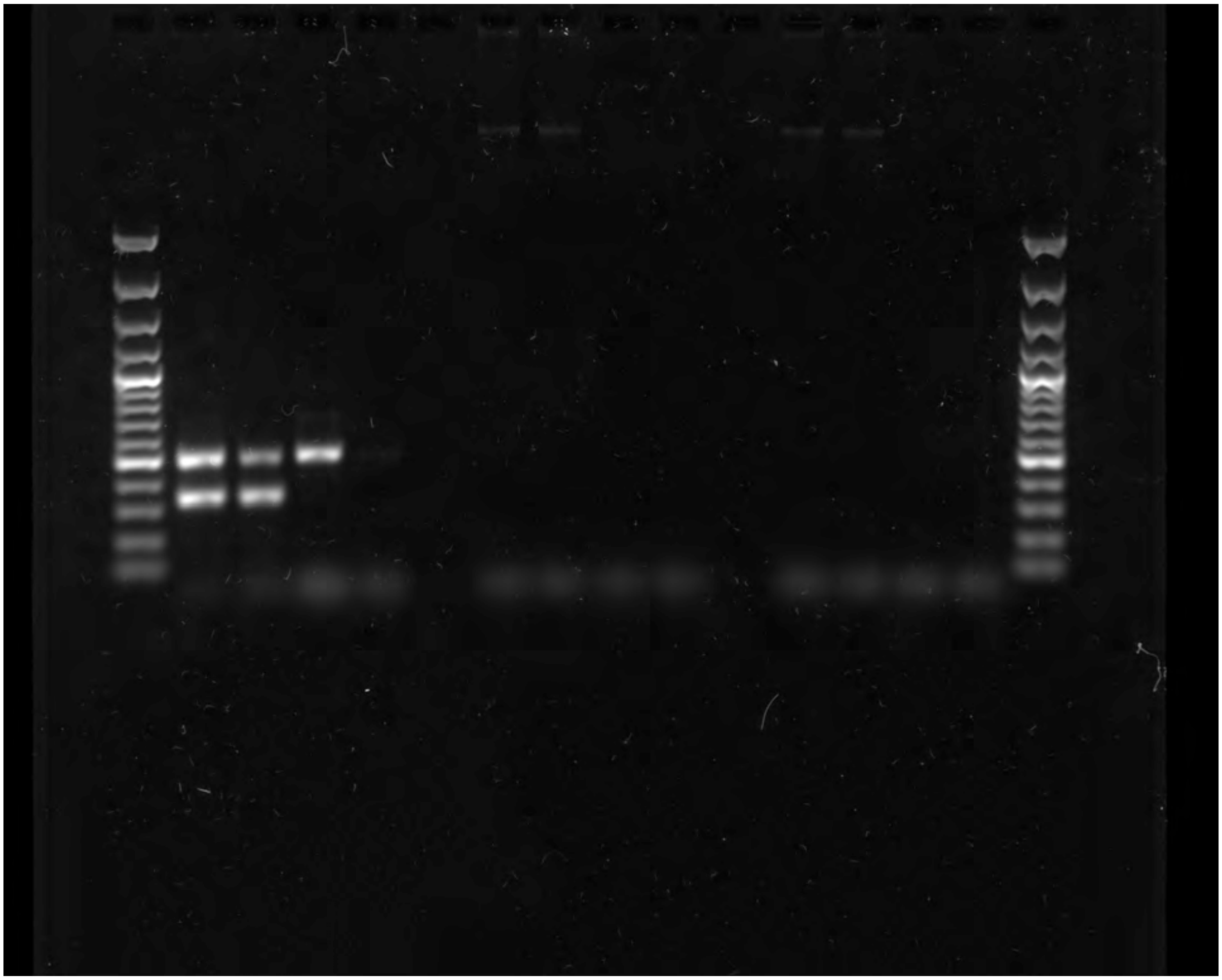

Figure S2

A

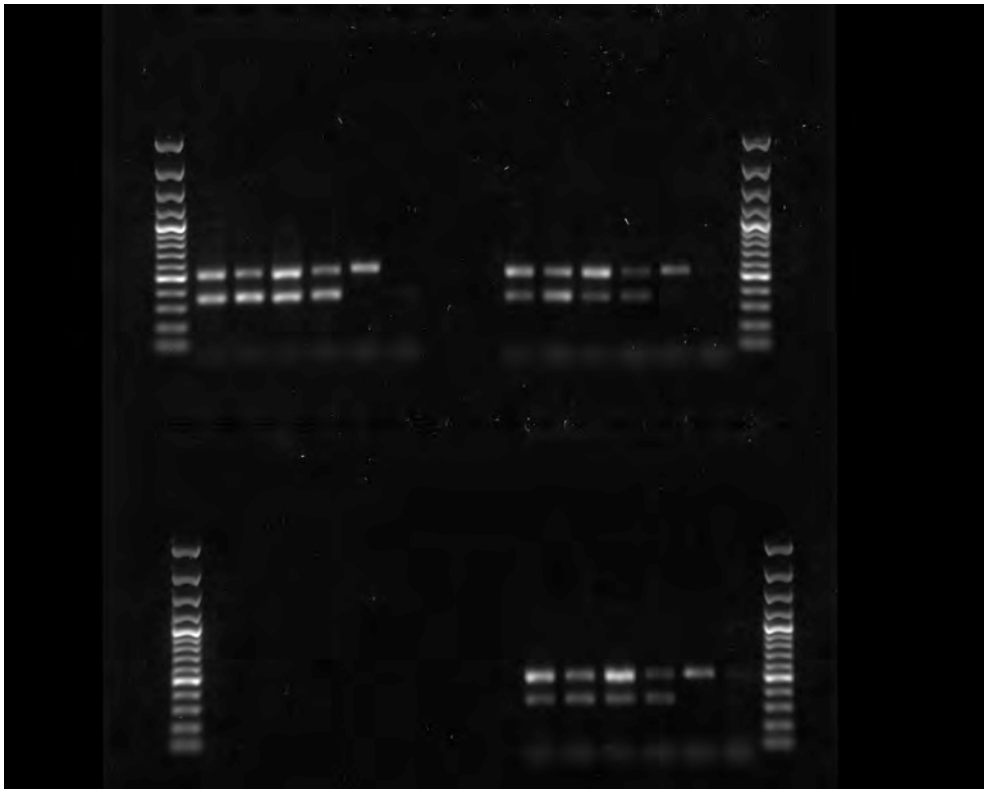

B (images of all 3 DNA samples)

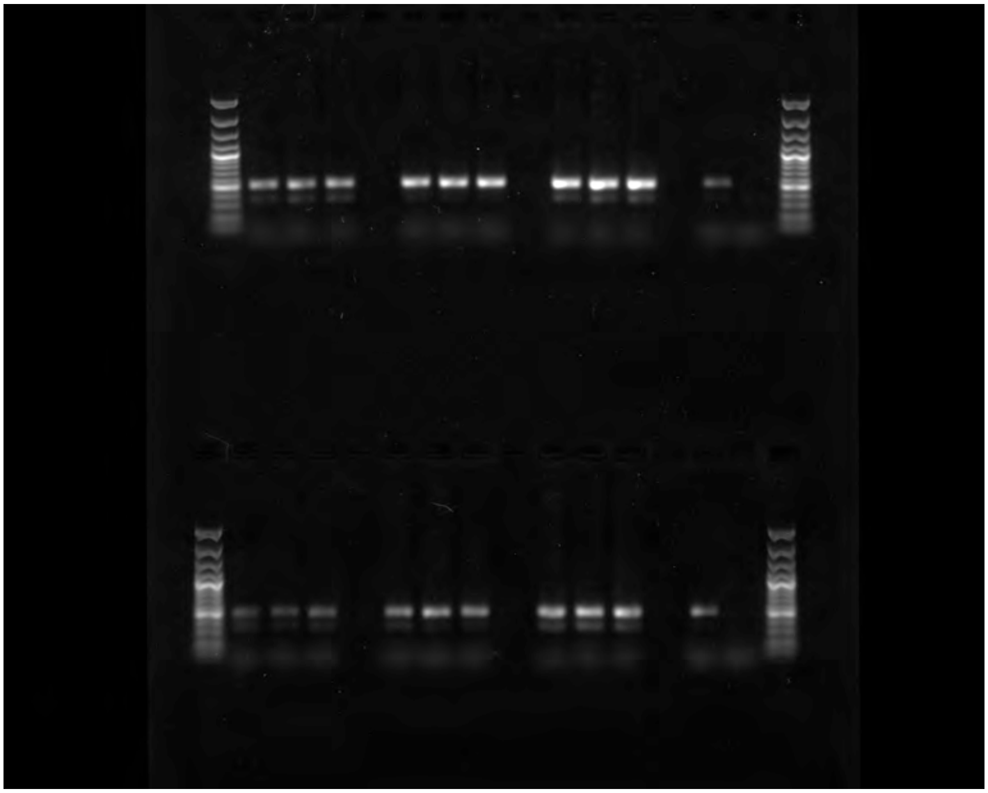

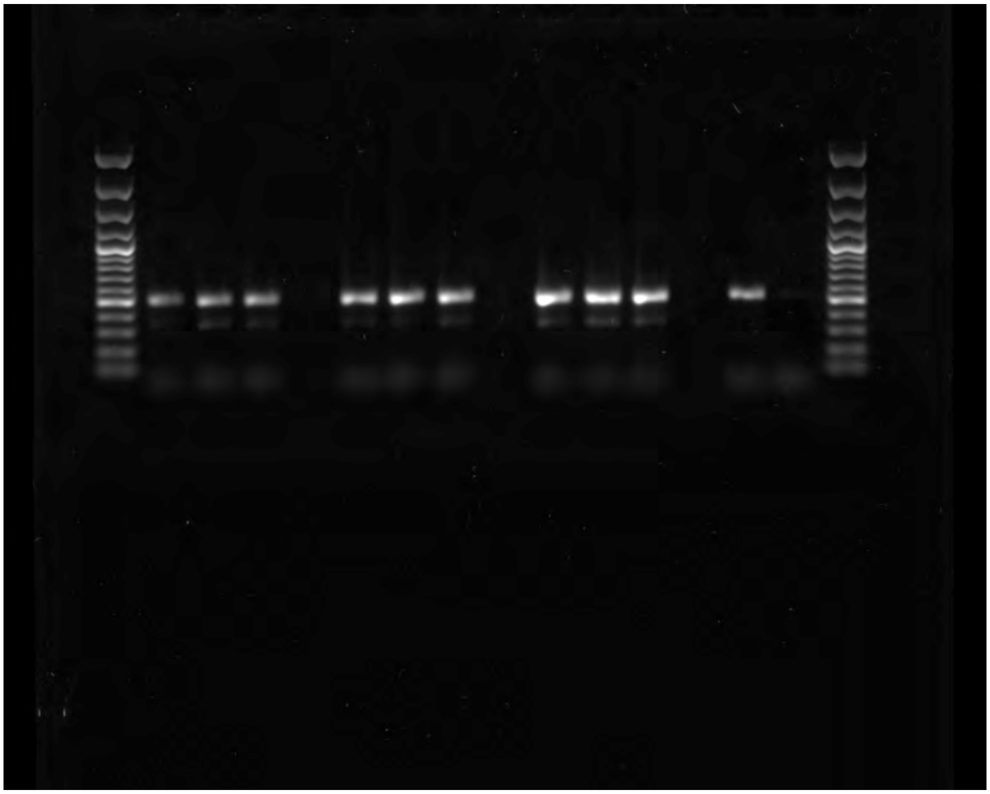

C

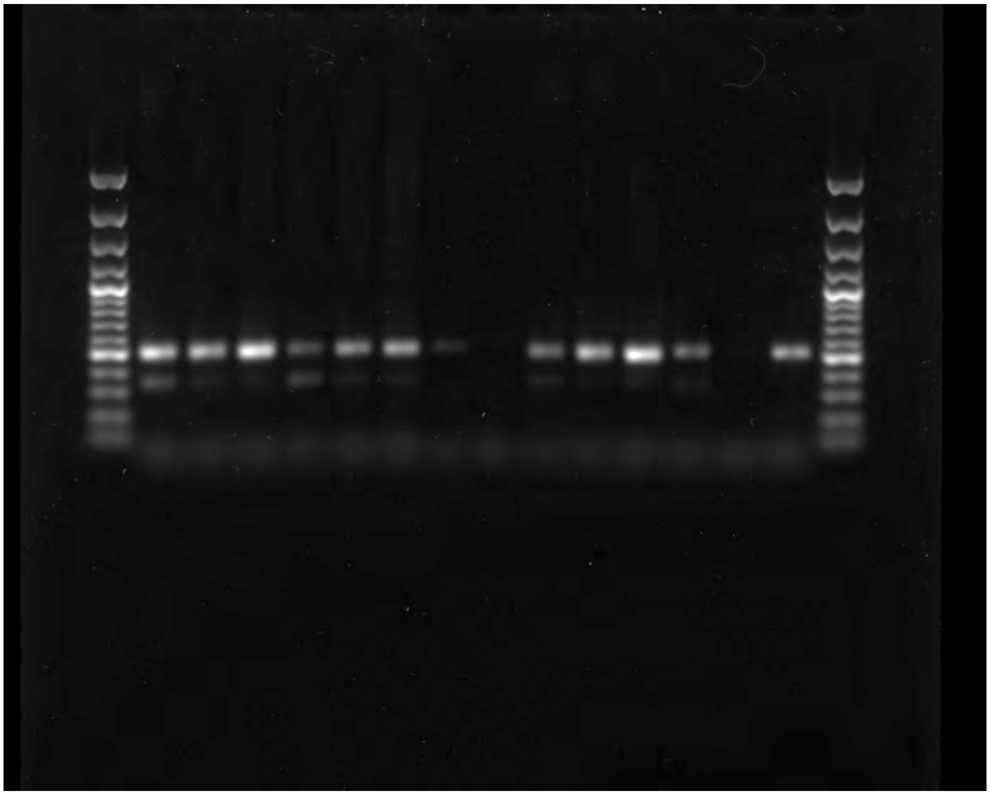

Figure S3

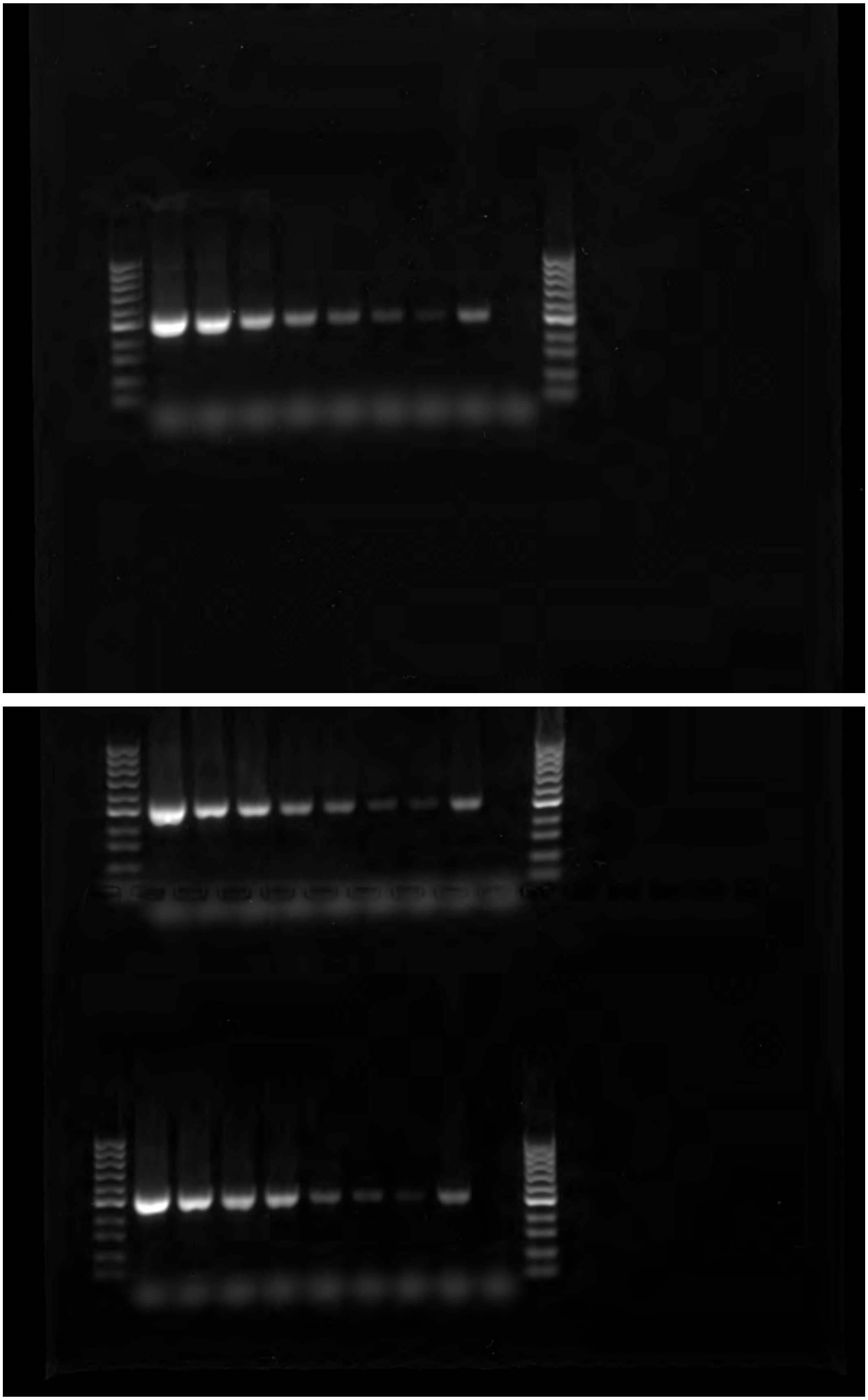

Figure S4

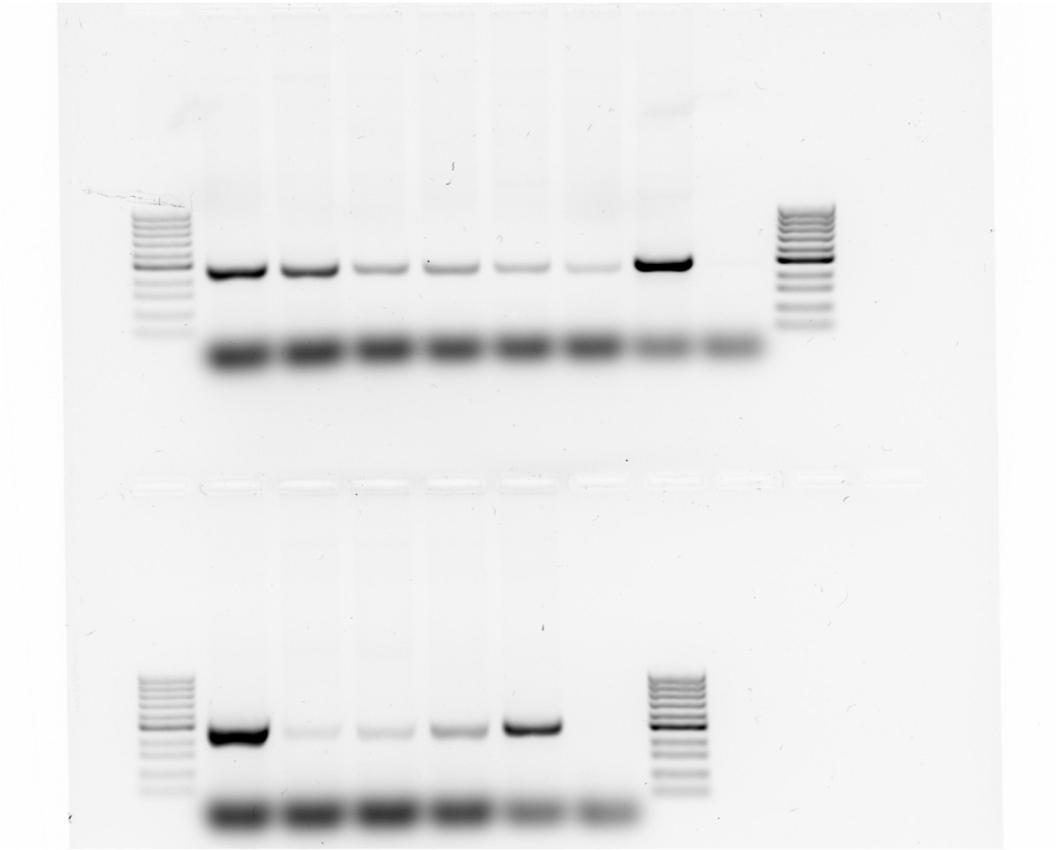

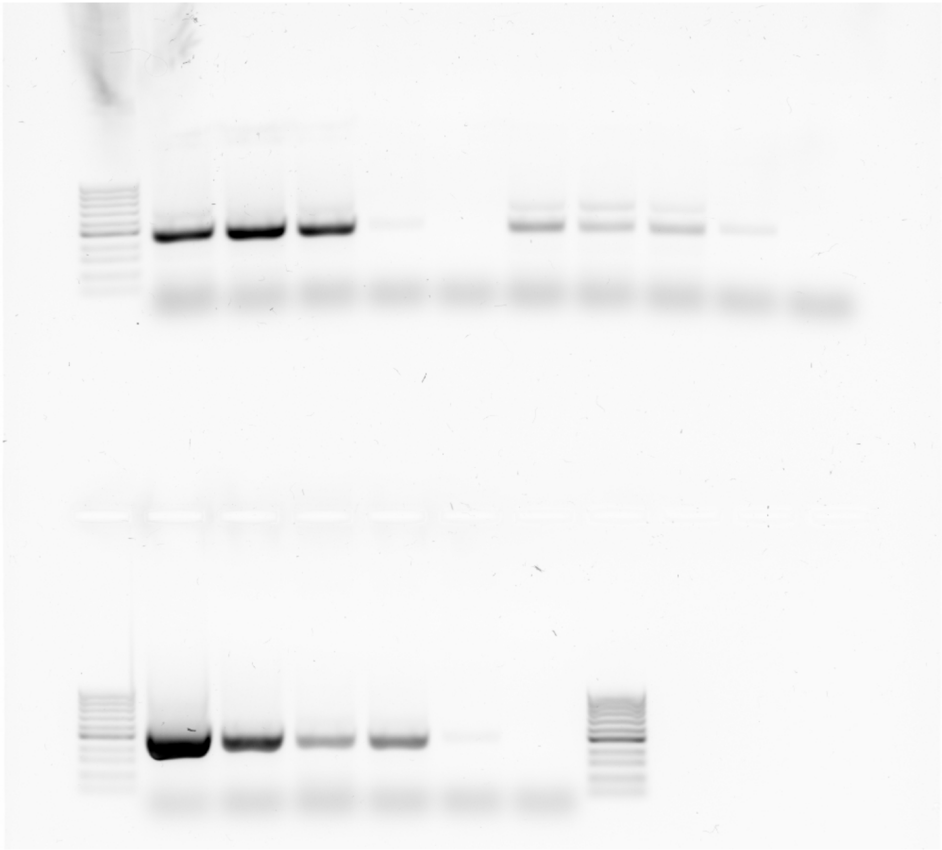

Figure S5

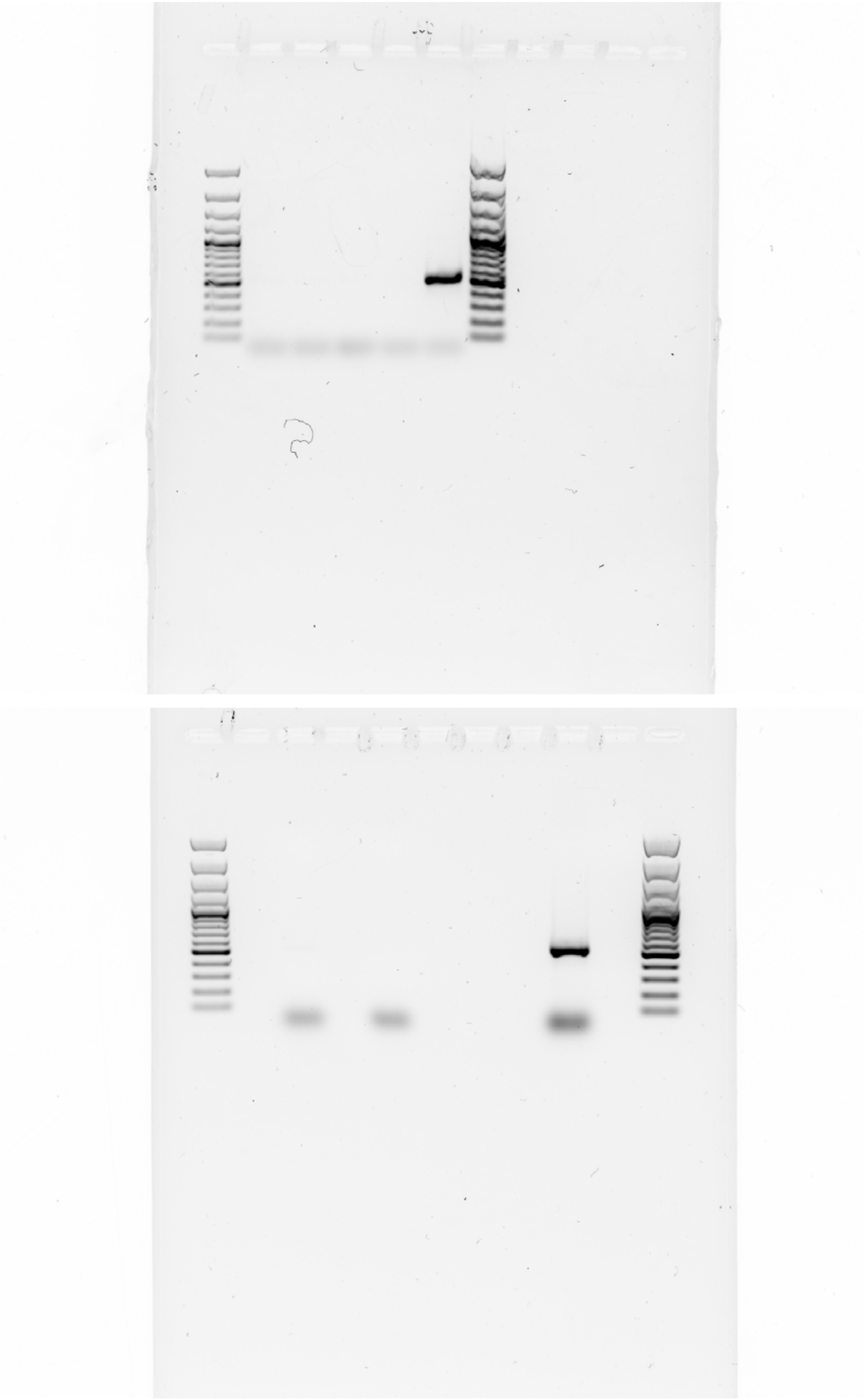

Supplement: Supplementary file 8 — Supplementary Material 8 [file 41598_2025_969_MOESM8_ESM.pdf]
