# Supplementary material for: Comparison of RNA- and DNA-based 16S amplicon sequencing to find the optimal approach for the analysis of the uterine microbiome
Source: Sci Rep. 2025 May 16;15:17037. doi: 10.1038/s41598-025-00969-5 (PMC12084623; doi:10.1038/s41598-025-00969-5)
Supplement: Supplementary file 9 — Supplementary Material 9 [file 41598_2025_969_MOESM9_ESM.pdf]

Supplementary file legends/information

Figure S1: Co-amplification of a mitochondrial 12S rRNA-derived product. **(A)** 16S rRNA V3-V4 amplicon PCR showing an additional amplification product at around 350 bp. S1/S2: DNA samples 1/2; P: positive control; N: negative control (H_2O). M: molecular weight standard (GeneRuler 100 bp Plus DNA Ladder). Agarose gel image shown as inverted greyscale. **(B)** Sanger sequencing AB1 trace file showing the result of sequencing the additional amplification product isolated from the agarose gel. At the bottom, the result of a BLAST search with the obtained sequence against NCBI nr sequence database (restricted to *Equus caballus*) is shown. **(C)** Illustration of the amplified sequence fragment of the equine mitochondrial 12S rRNA gene sequence with highlighted regions of Pro341F and Pro805R primer binding (primer sequences at the top, 12S rRNA sequence at the bottom). In addition, the sequence of the peptide nucleic acid (PNA) PCR clamp and its binding region in the 12S rRNA sequence (shown in red) as well as of the 12S rRNA blocking oligonucleotide and its binding region (underlined) are shown.

Figure S2: Suppression of the equine mitochondrial 12S rRNA amplification product. **(A)** Peptide nucleic acid (PNA) PCR clamp targeting the equine 12S rRNA gene. Control: PCR without PNA; 0.5 µM: PNA with 0.5 µM final concentration; 1 µM: PNA with 1 µM final concentration. Reactions for each group were performed with the same four cytobrush DNA samples. **(B)** 12S rRNA gene blocking oligonucleotide with 3‘-amino or -phosphate. Control: PCR without blocking oligonucleotide. Final concentration of blocking oligonucleotide was 0.2 µM. Reactions for each group were performed with the same three cytobrush DNA samples. **(C)** 12S rRNA gene blocking oligonucleotide with 3‘-amino with 0.2 and 0.4 µM final concentration. C: control PCR without blocking oligonucleotide. Reactions for each group were performed with the same three cytobrush DNA samples. P: positive control; N: negative control (H_2O). M: molecular weight standard (GeneRuler 100 bp Plus DNA Ladder).

Figure S3: Estimation of 16S rRNA V3-V4 amplicon PCR sensitivity. A serial dilution of the ZymoBIOMICS Microbial Community DNA Standard from 10 pg/µl to 0.15625 pg/µl was performed with three replicates (top, middle, bottom) and 1 µl used as template for 16S rRNA V3-V4 amplicon PCR. P: positive control; N: negative control (H_2O). M: molecular weight standard (GeneRuler 100 bp DNA Ladder).

Figure S4: Examples for DNA- and RNA-based 16S rRNA V3-V4 amplicon products. **(A)** DNA-based 16S rRNA gene V3-V4 amplicon PCR products for 10 cytobrush samples. **(B)** RNA-based 16S rRNA gene V3-V4 amplicon PCR products for the same 10 cytobrush samples as shown in A. N: negative control (H_2O). M: molecular weight standard (GeneRuler 100 bp DNA Ladder).

Figure S5: Controls for bacterial DNA contamination in kits and reagents. **(A)** Controls for water and buffer EB from Qiagen kit, and for PBS buffer. **(B)** Control for a fresh cytobrush processed for isolation of RNA and DNA. Of each cytobrush control sample for RNA and DNA isolation, 2 µl were used for cDNA and amplicon PCR, respectively. N: negative control (H_2O); P: positive control (ZymoBIOMICS Microbial Community DNA Standard, 5 pg input, 7.5 µl of 12.5 µl reaction loaded); M: molecular weight standard (GeneRuler 100 bp Plus DNA Ladder).

Figure S6: Taxonomic composition of individual DNA and RNA samples. Taxa with a relative abundance of >5% in at least one sample are shown for the taxonomic levels of class, order, and genus.

Figure S7: Identification of differentially abundant taxa between DNA- and RNA-based 16S rRNA analysis. Left: taxonomy bar plots showing differentially abundant taxa between DNA and RNA samples at phylum, class and order level. Right: box plots showing the relative abundance of differentially abundant taxa between DNA and RNA samples. Y-axis is in logarithmic scale (log 10).

Supplementary file Full images of agarose gel electrophoresis: Full images of agarose gel photos shown in Figures S1-S5.
