# Supplementary material for: Comparison of RNA- and DNA-based 16S amplicon sequencing to find the optimal approach for the analysis of the uterine microbiome
Source: Sci Rep. 2025 May 16;15:17037. doi: 10.1038/s41598-025-00969-5 (PMC12084623; doi:10.1038/s41598-025-00969-5)
Supplement: Supplementary file 10 — Supplementary Material 10 [file 41598_2025_969_MOESM10_ESM.docx]

Table S1. Quantification of 16S rRNA gene V3-V4 amplicon PCR products for a serial dilution of the ZymoBIOMICS Microbial Community DNA Standard from 10 to 0.15625 pg.

| Sample | pg DNA | Dilution factor | Vol R1 | Vol – N | Ratio to D1 | Ratio/dilution | Vol R2 | Vol – N | Ratio to D1 | Ratio/dilution | Vol R3 | Vol – N | Ratio to D1 | Ratio/dilution |
| --- | --- | --- | --- | --- | --- | --- | --- | --- | --- | --- | --- | --- | --- | --- |
| D1* | 10 | - | 382206565 | 359757455 | - | - | 318460383 | 300065000 | - | - | 306259263 | 293248550 | - | - |
| D2 | 5 | 2 | 224411643 | 201962533 | 1.8 | 1.8 | 187361697 | 168966314 | 1.8 | 1.8 | 216309772 | 203299059 | 1.4 | 1.4 |
| D3 | 2.5 | 4 | 176935528 | 154486418 | 2.3 | 1.3 | 147076118 | 128680735 | 2.3 | 1.3 | 148268287 | 135257574 | 2.2 | 1.5 |
| D4 | 1.25 | 8 | 101305327 | 78856217 | 4.6 | 2.0 | 84749055 | 66353672 | 4.5 | 1.9 | 122203978 | 109193265 | 2.7 | 1.2 |
| D5 | 0.625 | 16 | 67107540 | 44658430 | 8.1 | 1.8 | 56564952 | 38169569 | 7.9 | 1.7 | 40831988 | 27821275 | 10.5 | 3.9 |
| D6 | 0.3125 | 32 | 34495569 | 12046459 | 29.9 | 3.7 | 29510780 | 11115397 | 27.0 | 3.4 | 24393213 | 11382500 | 25.8 | 2.4 |
| D7 | 0.15625 | 64 | 31499609 | 9050499 | 39.8 | 1.3 | 26490723 | 8095340 | 37.1 | 1.4 | 17000140 | 3989427 | 73.5 | 2.9 |
| P |  |  | 79353566 | 56904456 |  |  | 67631396 | 49236013 |  |  | 54568445 | 41557732 |  |  |
| N |  |  | 22449110 | 0 |  |  | 18395383 | 0 |  |  | 13010713 | 0 |  |  |

D: dilution, P: positive control; N: negative control (used as background); Vol: volume; R: replicate; *software indicated band saturation
