# Supplementary material for: Comparison of RNA- and DNA-based 16S amplicon sequencing to find the optimal approach for the analysis of the uterine microbiome
Source: Sci Rep. 2025 May 16;15:17037. doi: 10.1038/s41598-025-00969-5 (PMC12084623; doi:10.1038/s41598-025-00969-5)
Supplement: Supplementary file 11 — Supplementary Material 11 [file 41598_2025_969_MOESM11_ESM.docx]

Table S2: Information for the mares used for collection of cytobrush samples

| No. | ID | Fertility | Breed | History | Cytology | Culture | K&D score^1^ | YOB^2^ |
| --- | --- | --- | --- | --- | --- | --- | --- | --- |
| 1 | E1_ES_02 | fertile | Arabian |  |  |  |  | 2016 |
| 2 | E1_ES_09 | fertile | Arabian |  |  |  |  | 2016 |
| 3 | E4_E_03 | PBIE | Standardbred | bred by live cover for 2 years | PMN | Strep. equi ssp. zoo. | IIB | 2008 |
| 4 | E4_E_05 | PBIE | Standardbred | Bred >3 times, not pregnant | PMN (3-5 pmn/hpf) | negative | IIB | - |
| 5 | E4_E_07 | PBIE | Quarter Horse | Bred 3 times, not pregnant | PMN (1-2 pmn/field) | negative | IIB | 2015 |
| 6 | E4_E_09 | PBIE | Quarter Horse | multiple attempts embryo transfer, maiden mare | PMN | E. coli | I | 2018 |
| 7 | E4_E_12 | PBIE | Standardbred | Pregnancy loss in 2021 | PMN (1-3 pmn/hpf) | negative | IIB | 2009 |
| 8 | E4_F_01 | fertile | Mixed breed | Fertile embryo transfer recipe | no PMNs | negative | I | 2006 |
| 9 | E4_U_01 | subfertile | Standardbred | Bred twice, maiden mare | no PMNs | negative | I | 2017 |
| 10 | E4_U_03 | subfertile | Standardbred | Bred >3 times | no PMNs | negative | IIB | 2012 |
| 11 | E4_U_05 | subfertile | Quarter Horse | Bred by live cover and AI for >2 years | no PMNs | negative | III | 2007 |
| 12 | E4_U_15 | subfertile | Mixed breed | Bred >3 times | no PMNs | negative | IIB | - |
| 13 | E4_U_16 | subfertile | Mixed breed | Bred >3 times | no PMNs | negative | IIB | - |
| 14 | E4_U_17 | subfertile | Mixed breed | Bred >3 times | no PMNs | negative | IIB | - |

^1^Kenney&Doig; ^2^year of birth
